# Transposon-colonized intron gain follows parasitism-mediated horizontal transfer of a cytochrome P450 gene

**DOI:** 10.1093/plphys/kiag335

**Published:** 2026-06-30

**Authors:** Eiichiro Ono, Kohki Shimizu, Jun Murata, Tenta Segawa, Akira Shiraishi, Ryusuke Yokoyama, Hiromi Toyonaga, Masaki Takagawa, Manabu Horikawa, Atsushi Hoshino, Koh Aoki

**Affiliations:** Research Institute, Suntory Global Innovation Center (SIC) Ltd., 8-1-1 Seikadai, Seika-cho, Soraku-gun, Kyoto 619-0284, Japan; Graduate School of Life and Environmental Sciences, Osaka Prefecture University, 1-1 Gakuen-cho, Naka-ku, Sakai, Osaka 599-8531, Japan; Bioorganic Research Institute, Suntory Foundation for Life Sciences (SUNBOR), 8-1-1 Seikadai, Seika-cho, Soraku-gun, Kyoto 619-0284, Japan; Research Institute, Suntory Global Innovation Center (SIC) Ltd., 8-1-1 Seikadai, Seika-cho, Soraku-gun, Kyoto 619-0284, Japan; Bioorganic Research Institute, Suntory Foundation for Life Sciences (SUNBOR), 8-1-1 Seikadai, Seika-cho, Soraku-gun, Kyoto 619-0284, Japan; Evolutionary Genomics, Graduate School of Life Science, Tohoku University, 6-3 Aramaki Aza-Aoba, Aoba-ku, Sendai, Miyagi 980-8578, Japan; Bioorganic Research Institute, Suntory Foundation for Life Sciences (SUNBOR), 8-1-1 Seikadai, Seika-cho, Soraku-gun, Kyoto 619-0284, Japan; Evolutionary Genomics, Graduate School of Life Science, Tohoku University, 6-3 Aramaki Aza-Aoba, Aoba-ku, Sendai, Miyagi 980-8578, Japan; Bioorganic Research Institute, Suntory Foundation for Life Sciences (SUNBOR), 8-1-1 Seikadai, Seika-cho, Soraku-gun, Kyoto 619-0284, Japan; Interdisciplinary Research Unit, National Institute for Basic Biology (NIBB), 38 Nishigonaka, Myodaiji, Okazaki, Aichi 444-8585, Japan; Graduate Institute for Advanced Studies, SOKENDAI (the Graduate University for Advanced Studies), 38 Nishigonaka, Myodaiji, Okazaki, Aichi 444-8585, Japan; Graduate School of Life and Environmental Sciences, Osaka Prefecture University, 1-1 Gakuen-cho, Naka-ku, Sakai, Osaka 599-8531, Japan; Graduate School of Agriculture, Osaka Metropolitan University, 1-1 Gakuen-cho, Naka-ku, Sakai, Osaka 599-8531, Japan

## Abstract

Specialized metabolites are often distributed sporadically across distantly related plant lineages, a pattern commonly attributed to convergent evolution, although the genomic processes enabling such innovation remain poorly understood. Here, we demonstrate that parasitic dodders (*Cuscuta* spp.) accumulate the lignan sesamin, a compound previously considered characteristic of sesame (*Sesamum indicum*) and related Lamiales species. We identified *Cuscuta* homologs of *S. indicum CYP81Q1*, which encodes piperitol/sesamin synthase (PSS), and demonstrated that these proteins retain catalytic PSS activity in vitro. Phylogenetic analyses indicate that *CYP81Q* was horizontally transferred from a Lamiales host to an ancestral *Cuscuta* lineage. Parasitism by *C. campestris* induces host *CYP81Q* expression and enhances interspecific transfer of genetic material across the haustorial interface, providing a mechanistic basis for horizontal gene transfer (HGT). Notably, comparative genomic analyses reveal that following horizontal acquisition, the transferred gene underwent extensive structural remodeling, characterized by sequential intron gains, while its enzymatic function was preserved. Many of the newly acquired introns exhibit hallmarks of insertion and excision of transposable elements, suggesting that mobile genetic elements contributed to post-transfer gene restructuring. The intron-rich architecture of *Cuscuta CYP81Q* was stably maintained throughout species diversification. Together, these findings suggest that parasitism-mediated HGT can be followed by intronization and transposon colonization, resulting in the generation of structurally complex yet functional genes. This process represents an underappreciated mechanism through which parasitic plants remodel horizontally acquired genes to facilitate metabolic innovation.

## Introduction

*Cuscuta* spp. (Convolvulaceae, Solanales), commonly referred to as dodders, are obligate parasitic plants with a broad host range (García et al. 2014; Costea et al. 2015). These plants possess limited or no photosynthetic capacity (Heide-Jørgensen 2008). To obtain the sugars and minerals required for survival and proliferation, *Cuscuta* has evolved the ability to form connections with autotrophic host plants through a specialized organ called a haustorium.

Despite their reduced photosynthetic capacity, *Cuscuta* spp. synthesize a diverse array of specialized metabolites, including flavonoids, steroids, alkaloids, and various other bioactive compounds. *Cuscuta* spp. have long been used in traditional Asian herbal medicine and have been reported to exhibit anti-aging, anti-inflammatory, hepatoprotective, analgesic, and aphrodisiac activities (Ahmad et al. 2017). The capacity to produce these metabolites may represent adaptive metabolic traits associated with parasitism and the unique physiological constraints imposed by a heterotrophic lifestyle.

**Figure 1:**
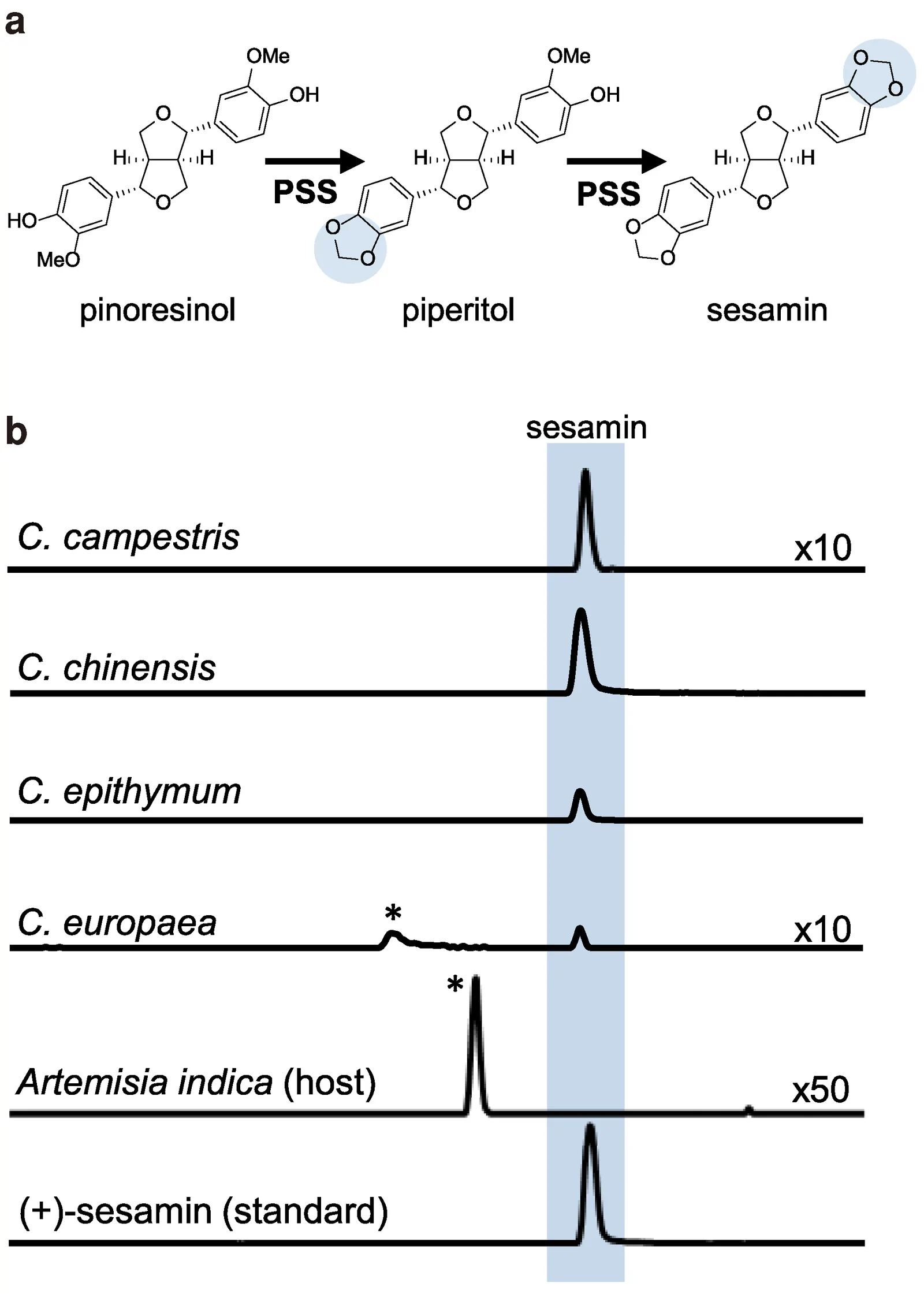
Accumulation of sesamin in *Cuscuta* species. (a) Biosynthetic pathway of sesamin catalyzed by piperitol/sesamin synthase, PSS, exemplified by *Sesamum indicum* CYP81Q1. (b) Extracted ion chromatograms (*m/z* 377.10, retention time 20–30 min) of *Cuscuta* extracts (top to bottom): floral clusters containing seeds of *C. campestris*, seeds of *C. chinensis*, seeds of *C. europaea*, stems of *C. epithymum*, leaves of Japanese mugwort (*Artemisia indica*), and an authentic (+)-sesamin standard. The (+)-sesamin peak is highlighted. Asterisks indicate unknown peaks. “×10” and “×50” denote vertical-axis magnification factors applied to the chromatograms.

Specialized metabolites are typically restricted to phylogenetically related taxa, known as chemotaxonomic groups, reflecting a shared biosynthetic origin inherited from a common ancestor. However, certain specialized metabolites occur sporadically across phylogenetically distant lineages, resulting in metabolic patchiness within otherwise coherent chemotaxonomic patterns. For example, sesamin, a specialized lignan, accumulates in the seeds of *Sesamum* spp. (sesame, Pedaliaceae, Lamiales) and in phylogenetically related plants such as Paulowniaceae, but has also been observed in distantly related taxa such as *Piper* spp. (black pepper), *Magnolia* spp. (basal angiosperms), and *Ginkgo* (a gymnosperm) (Srivastava et al. 2001; Umezawa 2003) (Figure S1). In *S. indicum*, a cytochrome P450 monooxygenase, SiCYP81Q1, also known as piperitol/sesamin synthase (PSS), catalyzes the sequential formation of 2 methylenedioxy bridges (MDBs) on the 2 aromatic rings of pinoresinol, converting pinoresinol to piperitol and subsequently to sesamin (Ono et al. 2006) (Fig. 1a). Recent studies indicate that accumulation of sesamin and its related metabolites in phylogenetic relatives of *S. indicum* coincides with the presence of functional *CYP81Q* genes that exhibit high sequence similarity to *SiCYP81Q1* (Ono et al. 2006; Noguchi et al. 2014; Ono et al. 2018). Notably, *Cuscuta* is, to date, the only genus in the Convolvulaceae family known to accumulate sesamin (Umezawa 2003; He et al. 2010; Abu-Lafi et al. 2018; Rho et al. 2018). The presence of sesamin in *Cuscuta chinensis, C. australis, and C. palaestina* suggests that they possess the biosynthetic enzyme required for sesamin production *in planta*. Since pinoresinol, the precursor of sesamin, is widespread across land plants, investigation of sesamin biosynthesis in *Cuscuta* spp. provides a model for understanding the molecular mechanisms that underlie the sporadic phylogenetic distribution of specialized metabolites.

Given that *PSS* genes in Lamiales currently represent the only known genes encoding proteins with sesamin synthase activity, the occurrence of sesamin in *Cuscuta* spp. implies the presence of a corresponding *PSS* gene in these parasitic plants. Because sesamin synthase activity has thus far been confined to a taxonomically restricted gene family, its presence in a phylogenetically distant lineage is difficult to explain by vertical inheritance alone and suggests the possibility of a gene acquisition event. Recent studies have demonstrated that parasitic plants are capable of acquiring genes from their host plants through horizontal gene transfer (HGT) (Zhang et al. 2014; Yang et al. 2016, 2019; Mariault et al. 2025). However, whether *Cuscuta* spp. have acquired a *PSS* gene via HGT, and whether such a gene retains biochemical functionality, remains unresolved.

In this study, we elucidated the molecular basis underlying the occurrence of sesamin and structurally related specialized lignans in *Cuscuta* spp. We identified homologs of *SiCYP81Q1* in multiple *Cuscuta* spp., all of which encode catalytically active PSS enzymes. In addition to demonstrating enzymatic functionality, comparative genomic analyses revealed that *Cuscuta* spp. acquired a functional *CYP81Q* gene from an ancestral sesamin-producing host, likely within Lamiales, through parasitism-mediated horizontal gene transfer (pHGT). Notably, intron gain, which is often associated with the insertion of transposable elements (TEs), was preferentially observed in the horizontally transferred *CYP81Q* genes, while catalytic activity was retained. This intron-acquired gene architecture was subsequently conserved and fixed during *Cuscuta* diversification, indicating stable functional integration of the transferred gene into the parasite genome.

## Results

### Identification of genes that show similarity to *SiCYP81Q1* in the genus *Cuscuta*

We analyzed lignans in seeds of *C. campestris* (Figure S2a), a member of the subgenus *Grammica*, parasitizing the experimental host plant *Nicotiana tabacum*. Sesamin and its related lignans were detected in the floral cluster of *C. campestris* (Fig. 1b, Figure S3a, b), but these compounds are not reported for *Nicotiana* spp. in the KNApSAcK metabolite database (Afendi et al. 2012). *Artemisia indica*, that is one of the preferred natural host plants of *C. campestris*, did not produce sesamin (Fig. 1b). These observations indicate that sesamin is not produced by host plants but is synthesized by *C. campestris*. Sesamin was also detected in *C. chinensis* of the subgenus *Grammica* and in *C. europaea* and *C. epithymum* of the subgenus *Cuscuta* (Fig. 1b, Figure S2b-d). These results also suggest that sesamin is synthesized de novo in *Cuscuta* plants.

**Figure 2:**
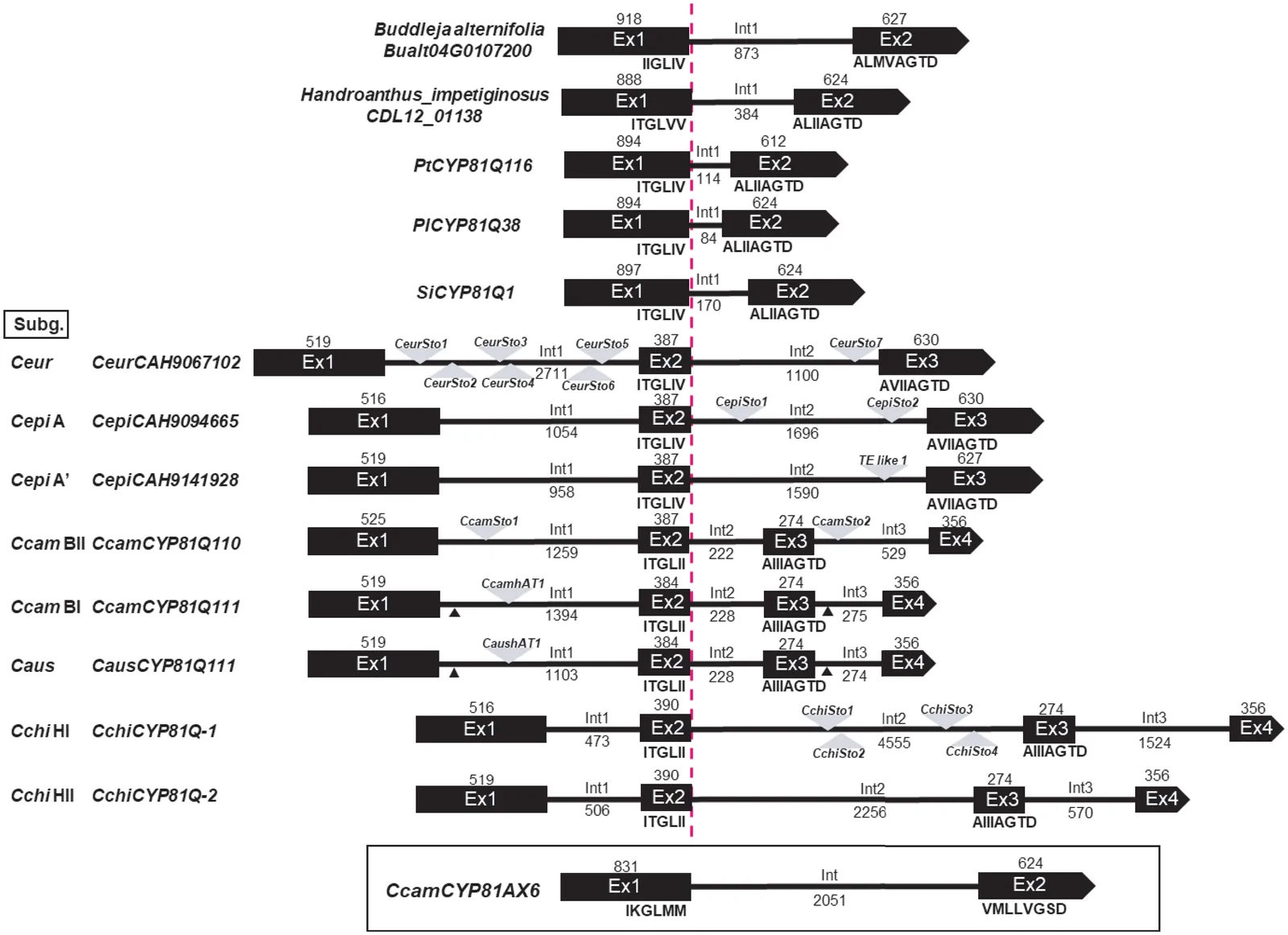
Genomic structures of CYP81Q genes. Exon-intron structures of CYP81Q genes from Lamiales species (Buddleja_alternifolia_BUALT_Bualt04G0107200, Handroanthus_impetiginosus_CDL12_01138, Paulownia tomentosa PtCYP81Q116, *Phryma leptostachya* PlCYP81Q38, and Sesamum indicum SiCYP81Q1) are aligned at the exon 1–intron 1 (Ex1–Int1) junction, and those from Cuscuta species are aligned at the exon 2–intron 2 (Ex2–Int2) junction. Amino acid sequences at the 3′-end of exon 1 or exon 2, and the 5′-end of exon 2 or exon 3 are shown. Ex: exon, Int: intron. Numbers indicate nucleotide lengths (bp). Gray triangles indicate transposons. Small black triangles indicate transposon remnants. CcamCYP81AX6 has an exon-intron structure distinct from that of CYP81Q genes. Cuscuta subgenomes (Subg.) onto which each gene is localized are shown in the left. Subgenome IDs are as in the Figure S5.

**Figure 3:**
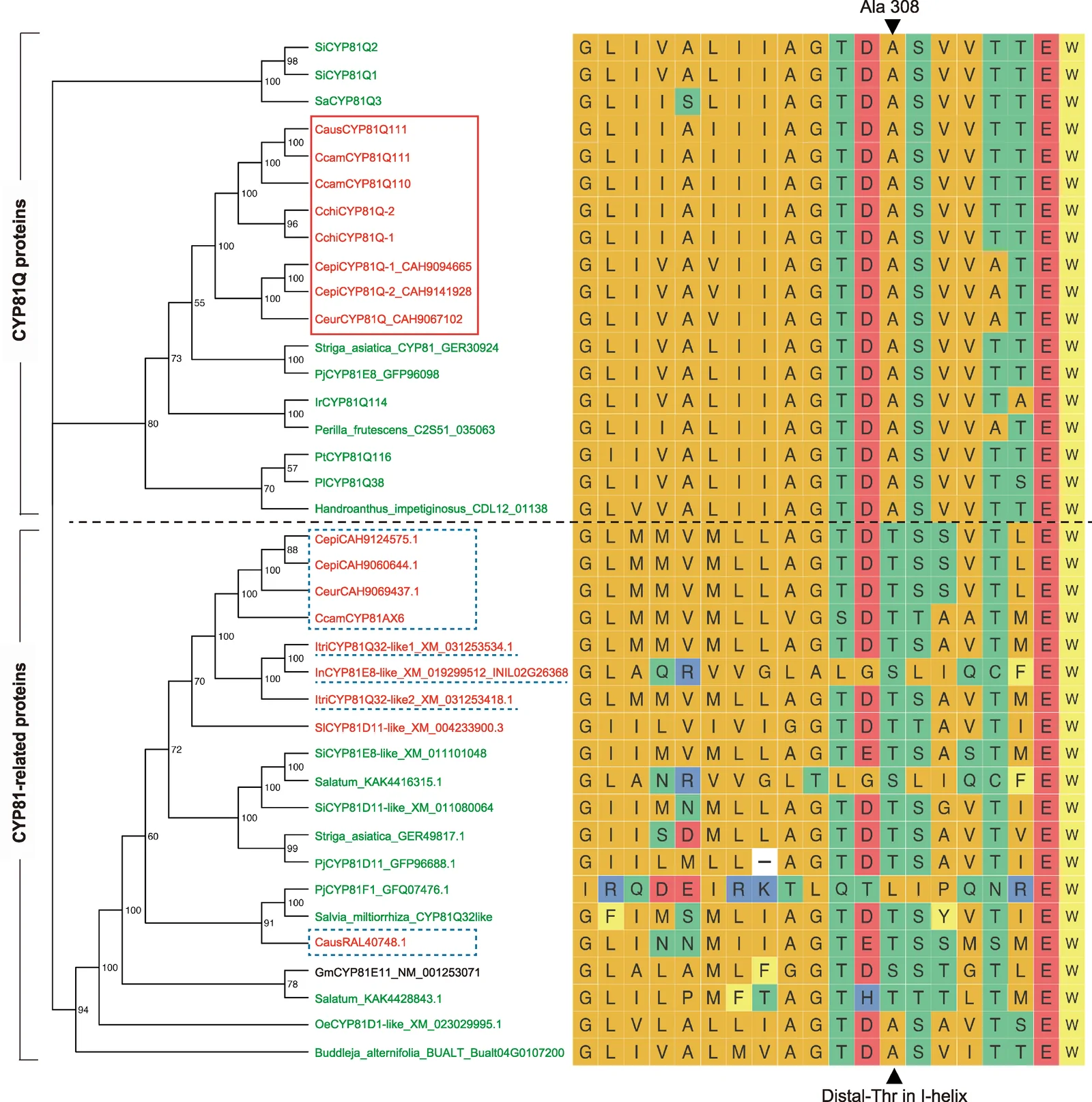
Phylogeny of CYP81Q proteins and CYP81-related proteins. (Left) Maximum likelihood phylogenetic tree of CYP81Q proteins (upper) and CYP81-related proteins (lower) in plants belonging to the orders Solanales, Lamiales, and Fabales. CYP81-related proteins were included as outgroups in the phylogenetic analysis of CYP81Q proteins. Protein IDs are provided in Table S1. Numbers on branches represent bootstrap support values. Taxonomic order of each plant species; black: Fabales, green: Lamiales, orange: Solanales. Orange box: Cuscuta CYP81Q proteins. Blue dotted box: Cuscuta CYP81-related proteins from different subfamilies. Blue dotted lines: *Ipomoea* proteins with the highest similarity to SiCYP81Q1 in each species. (Right) Alignment of amino acid sequences surrounding the distal-Thr residue in the I-helix. Triangles indicate the position of the conserved alanine in the CYP81Q proteins (Ala 308) and the conserved threonine in the CYP81-related proteins (Distal-Thr in I-helix).

To elucidate the molecular basis of sesamin biosynthesis in *Cuscuta* species, we searched for genes that exhibit similarity to *SiCYP81Q1* across multiple *Cuscuta* genome assemblies (GCA_003260385.1, GCA_900332095.2, GCA_945859875.1, and GCA_945859915.1) (Sun et al. 2018; Vogel et al. 2018) using the Basic Local Alignment Search Tool (BLAST) (Altschul et al. 1990). We identified genes that display high sequence similarity to *SiCYP81Q1* (hereafter referred to as *CYP81Q* genes) in species belonging to the subgenera *Grammica* and *Cuscuta*, and named them as *CausCYP81Q111* in *C. australis*; *CcamCYP81Q110* and *CcamCYP81Q111* in *C. campestris*; *CchiCYP81Q-1* and *CchiCYP81Q-2* in *C. chinensis*; *CeurCYP81Q_*CAH9067102.1 in *C. europaea*; and *CepiCYP81Q-1_*CAH9094665.1 and *CepiCYP81Q-2*_CAH9141928.1 in *C. epithymum* (Figs. 2 and 3 and Table S1). The predicted amino acid sequences of these genes share 78% to 95% identity with SiCYP81Q1 (Table S2).

### Genomic structure of the *Cuscuta CYP81Q* genes

*SiCYP81Q1* consists of 2 exons separated by a short single intron (Fig. 2, Ono et al. 2018). By contrast, we found that *Cuscuta CYP81Q* genes contain both a greater number of introns and substantially increased intron lengths compared with intron 1 of *SiCYP81Q1* (Fig. 2). *CYP81Q* from species of the subgenera *Cuscuta* and *Grammica* contain 2 and 3 introns, respectively. In addition, the position of the second intron in *Cuscuta CYP81Q* genes corresponds exactly to that of the intron position in *SiCYP81Q1*. Conservation of intron position between these phylogenetically distant lineages, ie, between *Cuscuta* and *Sesamum,* suggests a shared evolutionary origin of the genes. In all *Cuscuta CYP81Q* genes, the conserved lengths of the exon 1 and 2 suggest that intron 1 was inserted at nearly the same position, thereby splitting the original exon 1 of *CYP81Q* genes in Lamiales species (Fig. 2). Similarly, intron 3 of the *CYP81Q* genes in the subgenus *Grammica* was inserted at the same location, splitting the original exon 2 of these genes in Lamiales species (Fig. 2).

### *Cuscuta* genes belonging to *CYP81Q*- and other *CYP81* subfamilies

The position and the size of exon 2 are conserved among *Cuscuta CYP81Q* genes (Fig. 2). We analyzed exon 2 sequences of *CYP81Q* genes from other *Cuscuta* species, including *C. americana*, *C. californica*, and *C. gronovii*, for which whole-genome sequences are not yet available. Phylogenetic analyses incorporating these sequences (Table S3) together with data from the 8 sequenced species were consistent with the established phylogeny of *Cuscuta* spp. (García et al. 2014), supporting a monophyletic origin of these genes within the diversification of *Cuscuta* species (Figure S4).

The amino acid sequence of SiCYP81Q1 contains a unique Ala residue (Ala308) in the distal I-helix that is essential for the catalysis of MDB formation. In most cytochrome P450 enzymes, this position is normally occupied by a conserved Thr residue (the distal-Thr) (Fig. 3 and Table S4) (Imai et al. 1989; Noguchi et al. 2014). *CYP81Q* genes in *C. australis*, *C. campestris*, *C. chinensis*, *C europaea*, and *C. epithymum* all possess an Ala residue at the position corresponding to the distal-Thr residue (Fig. 3). This conservation of a crucial residue for catalysis satisfies the structural criteria for putative MDB-forming enzymes and suggests that these proteins likely catalyze sesamin biosynthesis.

In addition to *CYP81Q* genes, we identified *CcamCYP81AX6*, which exhibits a lower sequence similarity to *SiCYP81Q1* in *C. campestris*. Amino acid identity of CcamCYP81AX6 protein with SiCYP81Q1 is 51% (Table S2). CcamCYP81AX6 protein does not have Ala, but retains Thr in the distal-Thr position. We also identified homologs of *CcamCYP81AX6* in *C. australis*, *C. europaea*, and *C. epithymum*. Phylogenetic analysis showed that *CcamCYP81AX6* homologs form a distinct clade with *CYP81D/E*-related genes from *I. nil*, *I. triloba*, and several Lamiales species, with a single gene from *C. australis* (CausRAL40748.1) constituting an exception (Fig. 3, Table S1 and S4). This clade is distinct from the cluster containing *Cuscuta CYP81Q* genes (Fig. 3).

### *CYP81Q* genes and *CYP81*-related genes in Lamiales and Solanales

To clarify the distribution of *CYP81Q* genes, we investigated whether other species within the Lamiales and Solanales harbor *CYP81Q* genes (Fig. 3, Table S1 and S4). Species belonging to Lamiales, such as *Buddleja alternifolia*, *Phryma leptostachya*, *Paulownia tomentosa*, *Phtheirospermum japonicum*, *Striga asiatica*, *Isodon rubescens*, and *Perilla frutescens*, all harbor *CYP81Q* genes. The *CYP81Q* genes of these Lamiales species consist of 2 exons separated by a single intron (Fig. 2). The CYP81Q proteins of these Lamiales species uniformly possess an Ala residue at the site corresponding to the distal-Thr residue (Fig. 3). *CYP81Q* genes from *Cuscuta* species are nested within the same phylogenetic clade as these Lamiales *CYP81Q* genes, supporting their close evolutionary relationship.

On the other hand, although *Ipomoea* represents the closest relative of *Cuscuta*, no *CYP81Q* genes were identified in the genomes of *Ipomoea* species. *Ipomoea triloba* (assembly: GCA_003576645.1) (Wu et al. 2018) contains 2 genes, XM_031253418.1 and XM_031253534.1, and *I. nil* (assembly: GCA_001879475.1) (Hoshino et al. 2016a) contains one gene (XM_019299512.1) that exhibits moderate sequence similarity to *SiCYP81Q*1. However, none of these genes encode a protein with an Ala at the site corresponding to the distal-Thr residue. Similarly, *S. lycopersicum* (assembly: GCA_000188115.4) (Tomato Genome Consortium 2012) contains a single gene (XM_004233900.3) with sequence similarity to *SiCYP81Q1*, but this protein also retains a Thr at the corresponding site. Phylogenetically, these genes clustered together with *CcamCYP81AX6* homologs from *Cuscuta* spp. (Fig. 3).

### Comparison of gene synteny supports the horizontal transfer of the *CYP81Q* gene

**Figure 4:**
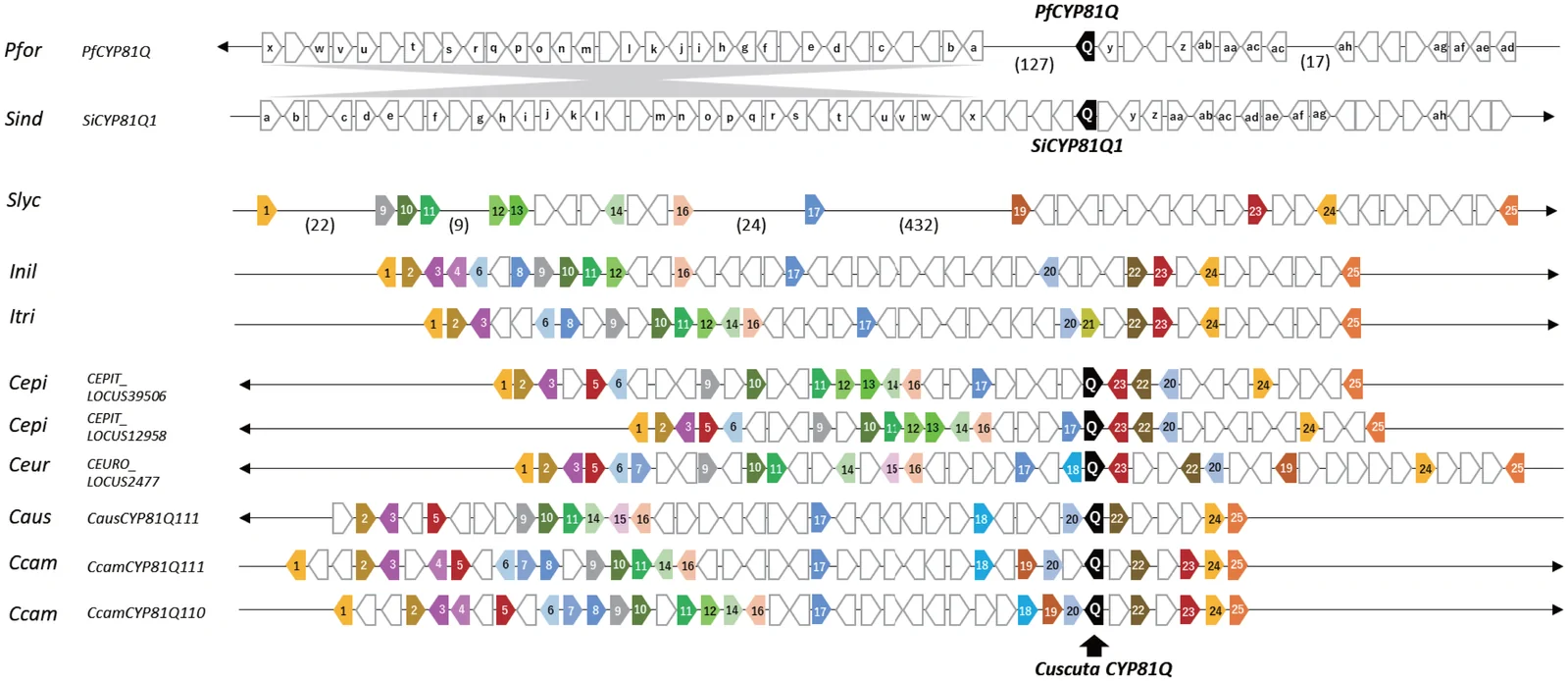
Comparative genomic synteny among *Cuscuta* and related species. Gene synteny in the genomic region surrounding *CYP81Q* genes is highly conserved among Solanales species, including *Cuscuta*, *Ipomoea*, and *Solanum*, whereas *Paulownia fortunei* and *Sesamum indicum* (Lamiales) show a distinct genomic structure. *Pfor*, *P. fortune; Sind*, *S. indicum; Slyc*, *Solanum lycopersicum; Inil*, *Ipomoea nil*; *Itri*, *I. triloba*; *Cepi*, *C. epithymum*; *Ceur*, *C. europaea*; *Caus*, *C. australis*; *Ccam, C. campestris*. Black boxes labeled “Q” indicate *CYP81Q* genes. IDs and descriptions of numbered genes are listed in Table S5. Numbers in parentheses indicate the number of genes within each interval. Gray triangles between *Pfor* and *Sind* denote chromosomal inversions.

We next compared gene synteny in the genomic regions harboring *CYP81Q* genes across *Paulownia fortunei*, *S. indicum, Cuscuta* species, and related genera within Solanales, including *Solanum* and *Ipomoea* (Fig. 4 and Table S5). In the *S. indicum* genome, *SiCYP81Q1* is located on chromosome 9 between genes encoding an FLX-like protein and an MYB44-like transcription factor. In *P. fortunei* (Lamiales), *PfCYP81Q* is found within a conserved syntenic block shared with *S. indicum*. In contrast, *CYP81Q* genes in *Cuscuta* species are embedded within a distinct genomic context (gene labels 17–25). Within *Cuscuta*, the relative order of 3 neighboring genes, gene labels 20, 22, and 23, was inverted between the subgenera *Cuscuta* and *Grammica*, whereas the surrounding upstream and downstream regions showed conserved synteny across all examined species (gene labels 1–12, 14, and 16). Comparable gene clusters with conserved gene order were also observed in *Ipomoea nil*, *I. triloba*, and *Solanum lycopersicum*. Notably, no genes corresponding to this conserved Solanales syntenic block were detected near *SiCYP81Q1* in *S. indicum*.

Together, these findings show that *Cuscuta CYP81Q* genes are embedded in a conserved Solanales syntenic block that is absent from the *S. indicum* genome, suggesting that *Cuscuta CYP81Q* was horizontally transferred from an ancestral Lamiales species into a genomic region where gene synteny is conserved within Solanales, rather than having arisen through gradual divergence from a Solanales ancestor. The HGT event is inferred to have occurred between 50.38 and 32.05 million years ago (Mya), after the divergence of parasitic *Cuscuta* from non-parasitic *Ipomoea* spp. and before the split between the subgenera *Cuscuta* and *Grammica* (Segawa et al. 2026), and well after the divergence of *Cuscuta* from *Sesamum* (Lamiales), estimated at 101.63 Mya (Figure S5).

### Functional evaluation of *Cuscuta CYP81Q* genes

**Figure 5:**
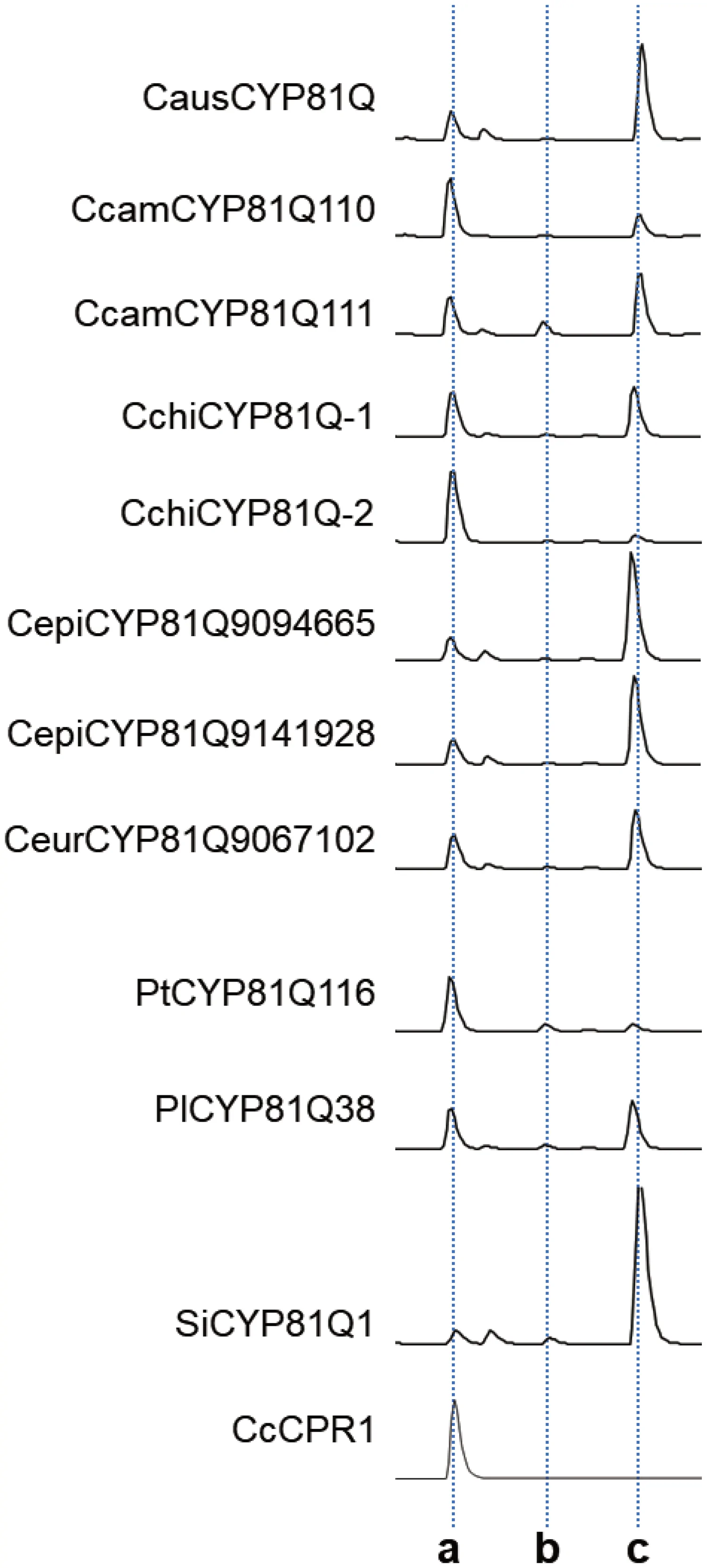
Enzymatic activity of *Cuscuta* PSS proteins. Products of yeast enzyme assays analyzed by HPLC. Chromatograms show absorption at 280 nm corresponding to lignan compounds. Names of the proteins tested are shown on the left; corresponding protein IDs are listed in Table S1. Peaks: a, (+)-pinoresinol (reaction substrate); b, (+)-piperitol; c, (+)-sesamin.

The co-occurrence of *CYP81Q* genes with sesamin and structurally related specialized lignans, such as cuscutoside, in *Cuscuta* species suggests that these genes function biochemically as PSSs. To evaluate the enzymatic activity of proteins encoded by *Cuscuta CYP81Q* genes, recombinant proteins were coexpressed in a yeast system together with the *C. campestris* cytochrome P450 reductase CcamCPR1 (VFQ62263.1) (Murata et al. 2017). When supplied with (+)-pinoresinol as a substrate, CausCYP81Q111, CcamCYP81Q110, CcamCYP81Q111, or CchiCYP81Q-1 each catalyzed the formation of 2 MDBs on the 2 aromatic rings of (+)-pinoresinol, yielding (+)-sesamin via the intermediate (+)-piperitol (Fig. 5). The stereochemistry of the resulting (+)-sesamin was identical to that generated by SiCYP81Q1 when (+)-pinoresinol was used as the substrate. In contrast, CcamCYP81AX6 did not exhibit MDB-forming activity toward any of the pinoresinol isomers tested (Figure S6). These results demonstrate that the 8 identified *Cuscuta CYP81Q* genes encode enzymatically active PSSs.

Analysis of publicly available RNA-seq datasets revealed that the 2 *CcamCYP81Q* genes and *CausCYP81Q111* were expressed in seedlings and flower bud clusters (Figure S7a and b) (Sun et al. 2018; Kaga et al. 2020). These expression patterns are consistent with the accumulation of sesamin in the flower buds of *C. campestris* (Fig. 1b). In contrast, *CcamCYP81AX6* showed negligible expression in these tissues.

Collectively, these results suggest that *Cuscuta* species synthesize sesamin through the activity of PSS enzymes encoded by *CYP81Q* genes, which are predominantly expressed in reproductive tissues.

### Parasitism-induced expression of *SiCYP81Q1* in hosts and its transfer to parasites

To explore the feasibility of pHGT of *CYP81Q*, we first tested whether *C. campestris* can parasitize *S. indicum*. *Cuscuta campestris* successfully established parasitic connections with sesame (Figure S8a and b) and subsequently developed flowers bearing fertile seeds (Figure S8c and d).

We next examined whether the inferred HGT event involved the transfer of a genomic DNA fragment encompassing an ancestral *CYP81Q* gene containing an intron. To test this possibility, we compared sequence similarity between genomic regions flanking *CYP81Q* genes in *Cuscuta* plants and *S. indicum*. Although substantial sequence similarity was detected between *CYP81Q* exon regions, the flanking regions showed extremely low similarity (Figure S9).

We then examined whether the acquisition of the *CYP81Q* genes could have been mediated by RNA transfer (Yoshida et al. 2010). Analysis of publicly available *S. indicum* RNA-seq datasets (Wang et al. 2014; Wang et al. 2015; Murata et al. 2017) revealed the presence of minor intron-retained (IR)-mRNA isoforms of *SiCYP81Q1*, which accounted for <3% of total transcripts in *S. indicum* (Figure S10). Both the fully spliced and IR forms of *SiCYP81Q1* were significantly induced in response to parasitism by *C. campestris* (Figure S8e to g), and IR-mRNA of *SiCYP81Q1* was detected in the stem of *C. campestris* (Figure S8h). These results indicate that *SiCYP81Q1* expression was induced in response to parasitism and that IR variants were translocated into *Cuscuta*, raising the possibility that RNA-mediated transfer contributed to the HGT of *CYP81Q*. However, whether such transferred transcripts can be stably integrated into the *Cuscuta* genome remains speculative.

### Enlarged introns colonized by transposons in *Cuscuta CYP81Q* genes

Interestingly, TEs and their footprints were detected within the enlarged introns, including frequent insertions of a nonautonomous DNA transposon of the *Mariner*/*Tc1* class, *Stowaway* (*Sto*), and *Sto*-derived footprints (Fig. 2, Table S6, and Figure S11). The highest sequence similarity was observed with *Sto* elements from *Ipomoea*, indicating that these elements likely originated from endogenous *Cuscuta* transposons rather than through horizontal transposon transfer (HTT) from host plants. This pattern supports the hypothesis that intron gain and expansion occurred after the HGT event in dodders. Importantly, we observed that the position of *Sto* elements within the *CYP81Q* introns of *C. europaea, C. epithymum, C. campestris, C. australis,* and *C. chinensis* varied among species, whereas the positions of a *hAT1* insertion and an associated *Sto* footprint within the *CYP81Q111* introns of *C. australis* and *C. campestris* exhibited notable similarity.

**Figure 6:**
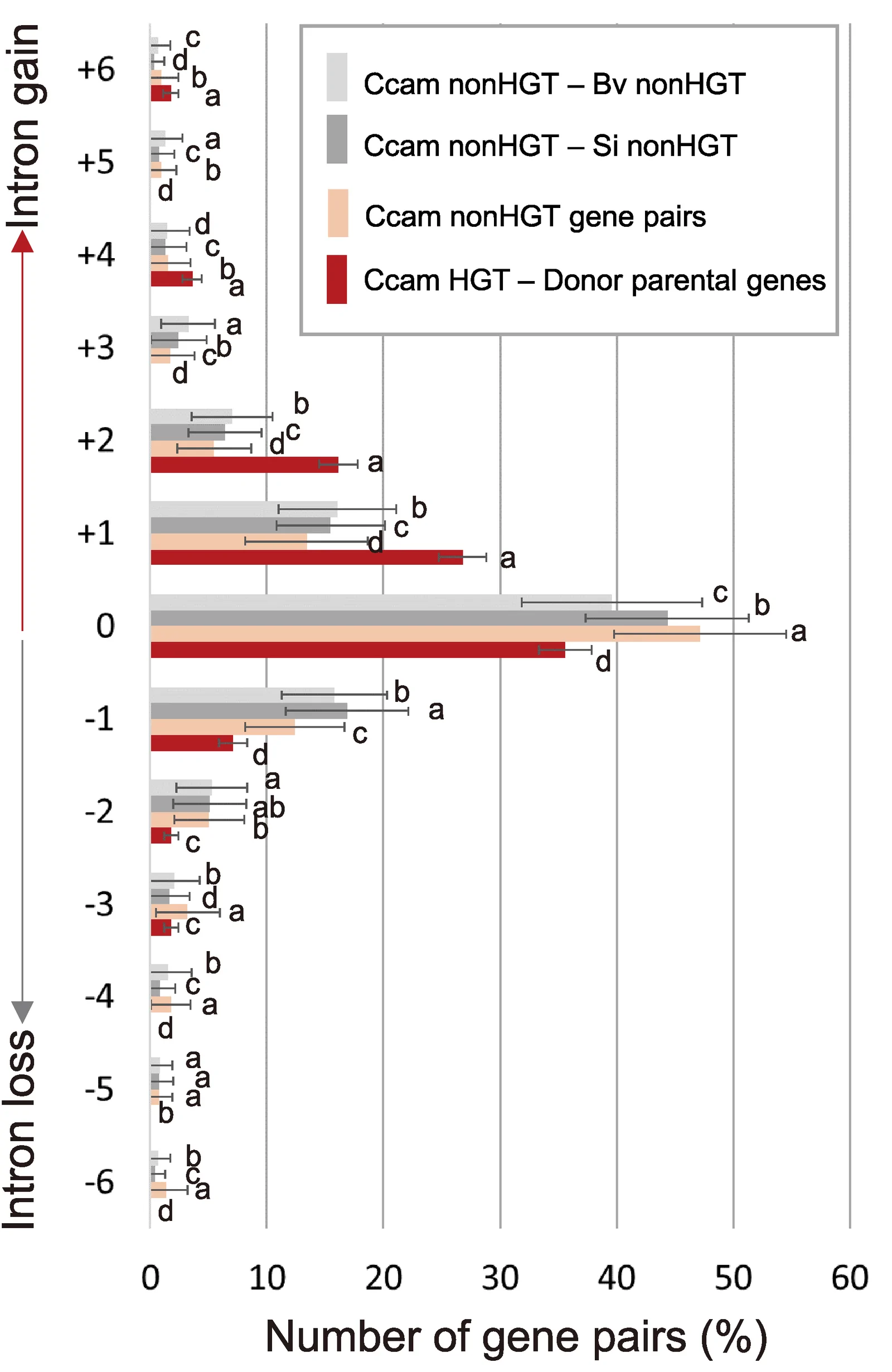
Changes in intron number in HGT genes. Distribution of differences in intron number per gene. The vertical axis shows the difference in intron number between the following gene pairs: Ccam nonhorizontal transfer (nonHGT) genes and *Beta vulgaris* nonHGT genes with the highest sequence similarity (nonHGT—Bv nonHGT); *Ccam* nonHGT genes and *S. indicum* nonHGT genes with highest similarity (Ccam nonHGT—Si nonHGT); *Ccam* nonHGT genes and their closest homologs (Ccam nonHGT gene pair); *Ccam* HGT genes and their orthologous genes from putative host species (Ccam HGT—Donor parental gene). The horizontal axis indicates the percentage of genes showing each level of intron number difference. Alphabetic letters above bars indicate statistically significant differences estimated by Tukey's honest significant difference test (*P* < 0.05).

In response to the observed intron gain in *Cuscuta CYP81Q* genes, we quantified intron numbers in 56 additional putative HGT-derived genes in *C. campestris* (Vogel et al. 2018). A high frequency of increased intron number increases (+1, +2, +4, +6) was detected in these HGT genes relative to their predicted donor orthologs (CcamHGT-donor parental genes; Fig. 6 and Table S7). Conversely, no significant increase in intron number was observed between paralogous non-HGT gene pairs within *C. campestris* (Ccam nonHGT gene pairs; Fig. 6 and Table S7). To exclude the possibility that these intron gains reflect intrinsic interspecies differences, we compared intron numbers between *C. campestris* and the predicted donor plants for orthologous gene pairs not derived from HGT of *Beta vulgaris* and *S. indicum* (Ccam nonHGT-Bv nonHGT, Ccam nonHGT-Si nonHGT; Fig. 6 and Table S7). Neither comparison revealed significant differences in intron number, indicating that the increased intron counts in HGT genes are not attributable to baseline genomic differences between donor and recipient plant genomes.

### Retention of *CYP81Q* is associated with metabolic innovation in *Cuscuta*

**Figure 7:**
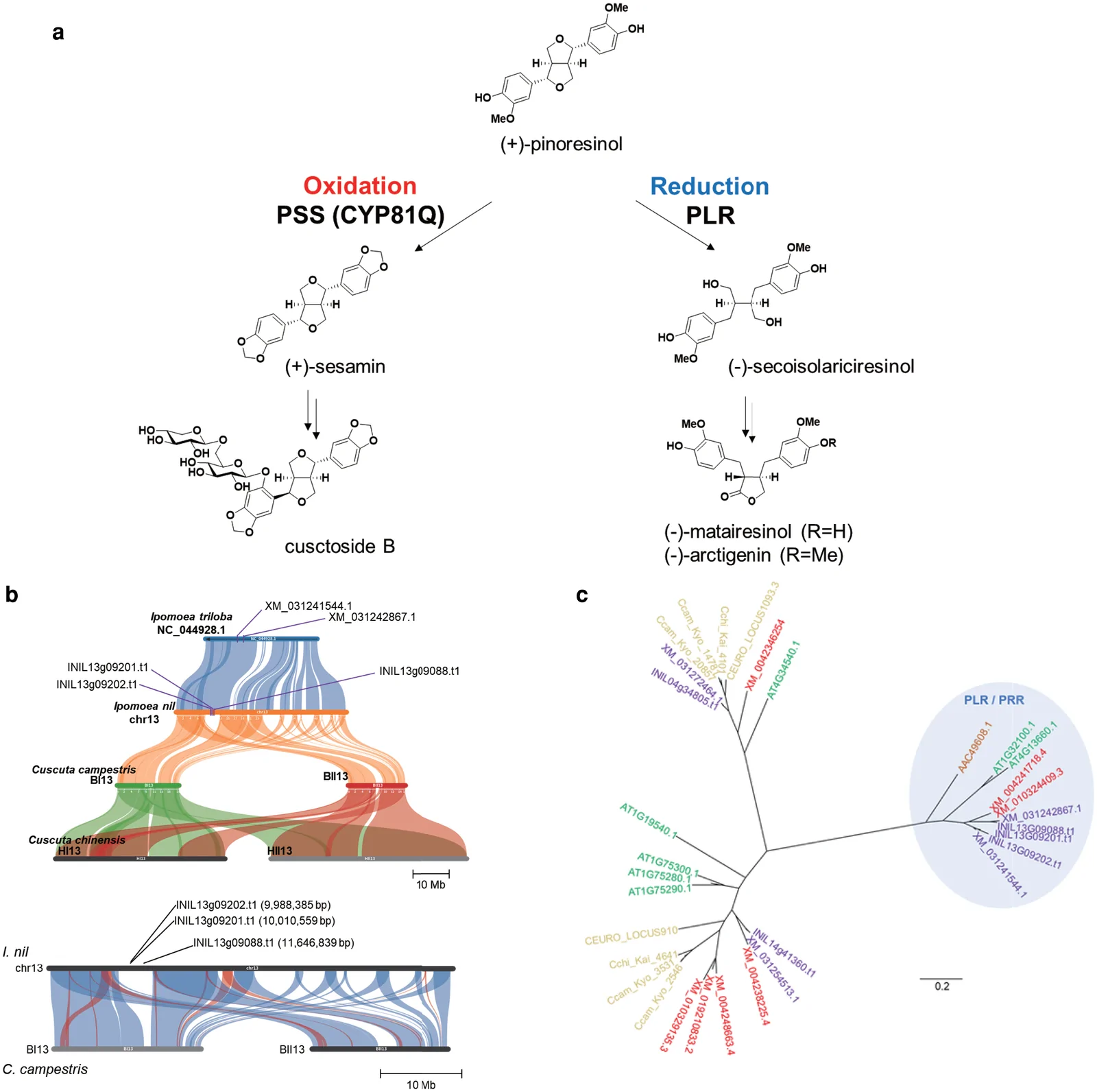
Gene synteny and phylogenetic analyses of pinoresinol lariciresinol reductase (PLR)-related genes. (a) Metabolic pathway of plant specialized lignans. Pinoresinol is the common substrate for PSS-catalyzed oxidative conversion to sesamin and PLR-catalyzed reduction to secoisolariciresinol. In *Cuscuta* spp., sesamin is further modified into cuscutoside through hydroxylation and glycosylation, while matairesinol and arctigenin are detected in *Ipomoea cairica*, suggesting functional PLR activity in the *Ipomoea* lineage and metabolic competition between PLR and PSS. (b) Gene synteny comparison between *Cuscuta* and *Ipomoea* species. The genomic region harboring Ipomoea *PLR-like* genes is absent in 2 *Cuscuta* species, despite high conservation of genomic synteny across most of the surrounding genomic region between these phylogenetically related taxa. (c) A Maximum-likelihood phylogenetic tree of *PLR-like* genes. Ccam, *C. campestris*; Cchi, *C. chinensis*, CEURO, *C. europaea*; INIL, *I. nil*; AT, *Arabidopsis thaliana*. XM_0042346254, XM_004238225.4, XM_004248663.4, XM_019210833.2, XM_010329135.3, XM_004241718.4, and XM_010324409.3 are from *Solanum lycopersicum*. XM_031272464.1, XM_031254513.1, XM_031242867.1, and XM_031241544.1 are from *Ipomoea triloba*. AAC49608.1 is from *Forsythia* × *intermedia.* No *Cuscuta* spp. homologs were recovered in the “PLR/PRR” clade including *FiPLR* and *Arabidopsis PINORESINOL REDUCTASE* (*PRR*) genes, suggesting that ancestral *Cuscuta* lacked *PLR-like* genes prior to speciation into *C. europaea.*

The metabolic context in which CYP81Q is maintained after pHGT was further investigated. To this end, we performed gene synteny and phylogenetic analyses of genes encoding pinoresinol/lariciresinol reductase (PLR). PLR competes with PSS for the common substrate pinoresinol and catalyzes its reduction to secoisolariciresinol rather than its oxidative conversion to sesamin (Fig. 7a). We found that genes encoding PLR are absent from *Cuscuta* species within the syntenic regions corresponding to those containing *Ipomoea PLR* genes (Fig. 7b). Furthermore, *Cuscuta* genes exhibiting sequence similarity to *PLR* form phylogenetic clades distinct from the clade containing *Ipomoea PLR*s and *Arabidopsis PINORESINOL REDUCTASES* (*PRRs*) (Fig. 7c). Given the reported metabolic competition between PLR and PSS for pinoresinol in engineered *Forsythia* species (Kim et al. 2009; Murata et al. 2015), the loss of *PLR* in the genus *Cuscuta* may have facilitated lignan metabolic flux toward sesamin and related metabolites following pHGT-mediated acquisition of PSS (Fig. 7a).

## Discussion

### Acquisition of sesamin synthase genes via pHGT in *Cuscuta*

Our results showed that a functional *CYP81Q* gene was horizontally acquired by an ancestral *Cuscuta* lineage from a Lamiales host capable of producing sesamin or related specialized lignans. The conserved capacity of CYP81Q proteins to catalyze MDB formation and generate specialized lignans suggests that this enzymatic activity confers a selective advantage in dodders. Although the overall frequency of functional pHGT events remains difficult to estimate, the long-term retention of *CYP81Q* genes across multiple *Cuscuta* species suggests strong selective pressure to maintain their function. Because most horizontally transferred genes are expected to be lost due to deleterious effects or lack of functional integration into endogenous metabolic networks, conserved, biochemically functional pHGT events such as *CYP81Q* likely represent a physiologically meaningful subset of successful transfers.

The estimated divergence between Lamiales and Solanales is approximately 101.63 Mya (Figure S5), whereas *Cuscuta* diverged from *Ipomoea* at approximately 50.38 Mya (Figure S5). These estimates indicate that the *CYP81Q* gene was acquired after the genus *Cuscuta* diverged from its non-parasitic relatives. In addition, the strict monophyly observed in *Cuscuta CYP81Q* genes (Figure S4) suggests that pHGT occurred once in the most recent common ancestor of the subgenera *Cuscuta* and *Grammica*, followed by vertical inheritance and lineage-specific diversification.

Consistent with this scenario, *CYP81Q* genes were identified in *C. campestris*, *C. australis*, *C. chinensis*, *C. epithymum*, and *C. europaea* (Figs. 2 and 3 and Table S2). These findings indicate that *CYP81Q* genes are broadly conserved within the subgenera *Grammica* and *Cuscuta*. As the occurrence of *CYP81Q* genes in *Monogynella* has not yet been determined, it remains unclear whether the gene was acquired before or after the divergence of *Monogynella* from the lineage leading to the subgenus *Cuscuta* (García et al. 2014). Comparative sequence analyses further suggest that, following speciation, *CYP81Q* genes accumulated nucleotide substitutions and lineage-specific modifications in exon–intron architecture. Notably, *CYP81Q* loci are localized within genomic regions exhibiting conserved synteny across *Cuscuta*, *Ipomoea*, and *Solanum* (Fig. 4), supporting their incorporation into the *Cuscuta* genome via pHGT. Although we cannot fully exclude the possibility that an ancestral *Ipomoea* species possessed a *CYP81Q* ortholog that was subsequently lost, this possibility is unlikely because, other than *Cuscuta*, no members of the Convolvulaceae are known to accumulate sesamin or related specialized lignans (Umezawa 2003; Afendi et al. 2012).

### Origin of sesamin synthase genes

In contrast to *Cuscuta* species, no homologs of *SiCYP81Q1* were identified in publicly available genome assemblies of *Piper nigrum*, *Magnolia ashei*, or *Ginkgo biloba*, despite documented sesamin biosynthesis in several phylogenetically disparate Lamiales lineages (Srivastava et al. 2001; Umezawa 2003; Ono and Murata 2023) (Figure S1). This absence is consistent with previous phylogenetic analyses indicating that cytochrome P450 family members are not conserved in basal angiosperms or gymnosperms (Nelson et al. 2004). Given the substantial evolutionary distance separating these taxa from Lamiales, the sesamin synthase genes responsible for lignan production in these species, which have yet to be identified, are unlikely to share a common evolutionary origin with *SiCYP81Q1* (Figure S1). Taken together, these observations suggest that the sporadic phylogenetic distribution of sesamin biosynthesis across the plant kingdom reflects the combined contributions of HGT and convergent evolution. In some lineages, such as *Cuscuta*, sesamin production appears to have been enabled by the HGT of *CYP81Q* genes, whereas in other lineages lacking *CYP81Q*, including *Magnolia*, *Ginkgo*, and *Piper*, sesamin biosynthesis is likely mediated by distinct, independently evolved oxygenases. The coexistence of these evolutionary routes provides a coherent explanation for the uneven distribution of sesamin production among plant lineages.

Within the genus *Cuscuta*, *CYP81Q* genes appear to have diversified in parallel with speciation events. This diversification coincides with an increase in genome size, which was previously attributed to a whole-genome duplication event estimated to have occurred approximately 1.5 Mya (Sun et al. 2018; Vogel et al. 2018). However, recent chromosome-level genome assemblies of *C. campestris* and *C. chinensis* instead indicate that these species originated through hybridization between closely related lineages via allopolyploidization (Segawa et al. 2026), which is consistent with earlier taxonomic hypotheses (Costea et al. 2015). Support for this hybrid origin is evident in sequence similarity patterns observed, where *CcamCYP81Q111* shows higher similarity to *CausCYP81Q111* than to *CcamCYP81Q110* (Fig. 3). Additionally, the distribution of TEs at the *CYP81Q* loci supports this observation. Specifically, *CcamCYP81Q111* and *CausCYP81Q111* share a *hAT1* in intron 1 and remnants of *Sto* in intron 3, while *CcamCYP81Q110* contains *Sto* in both introns 1 and 3 (Fig. 2). These results indicate that *CcamCYP81Q110* and *CcamCYP81Q111* constitute a pair of homoeologous genes derived from distinct parental lineages. Similarly, analyses of gene synteny, genomic divergence, and TE distribution indicate that *CchiCYP81Q-1* and *CchiCYP81Q-2* are also homeologs (Fig. 2, Segawa et al. 2026). Two *CYP81Q* genes were likewise identified in *C. epithymum*; however, the high sequence similarity between these genes, together with the limited availability of genome assemblies for other species in the subgenus *Cuscuta*, currently precludes a robust inference of a hybrid origin for *C. epithymum*. Future pan-genomic analyses incorporating additional chromosome-level assemblies will be essential for resolving the evolutionary histories of *CYP81Q* genes and for clarifying their broader phylogenetic relationships within *Cuscuta* (Neumann et al. 2021; Cerda-Herrera et al. 2025; Segawa et al. 2026).

Previously characterized plant HGT events have frequently been interpreted as involving the transfer of large genomic fragments (Kado and Innan 2018; Vogel et al. 2018). However, this mechanism appears unlikely for *CYP81Q* acquisition. Genomic regions flanking *CYP81Q* genes in *Cuscuta* exhibit extremely low sequence similarity to corresponding regions in *S. indicum* (Figure S9), suggesting that acquisition did not involve the transfer of a large DNA fragment. Instead, our observation that IR-mRNA isoforms of *SiCYP81Q1* are upregulated in *S. indicum* following parasitism and are detectable in *C. campestris* is consistent with RNA-mediated transfer contributing to pHGT (Figure S8e to h). Although the precise molecular mechanism involving *CYP81Q* acquisition remains unresolved, these findings suggest that locally abundant host transcripts may serve as substrates for horizontal transfer, potentially followed by genomic integration. Mechanisms proposed for HTT, which involve both DNA- and RNA-based intermediates, may therefore provide useful analogies for understanding how foreign genes become internalized in parasitic plant genomes (Schaack et al. 2010; Mariault et al. 2025).

### Transposon colonization in newly acquired introns of HGT genes

Following horizontal acquisition, the coding sequences of *Cuscuta CYP81Q* genes have gradually diverged (Fig. 3 and Table S4 and S8), and their genomic architectures have undergone lineage-specific rearrangement that broadly reflects the time elapsed since the HGT event. A prominent feature of this structural diversification is the acquisition of additional introns relative to the single intron present in ancestral Lamiales *CYP81Q* genes (Fig. 2). The position of the acquired intron 1 is highly conserved among *Cuscuta* species (Fig. 2), suggesting that the gain of this intron likely occurred early after the HGT event. Likewise, the conserved location of acquired intron 3 suggests that its gain may have occurred early in the speciation of the subgenus *Grammica*. To assess whether this pattern extends beyond *CYP81Q*, we compared intron numbers in 56 predicted HGT genes of *C. campestris* (out of a reported total of 73 genes) with those of their respective orthologs in putative host plants (Vogel et al. 2018). This analysis revealed a significant increase in intron number among HGT genes in *C. campestris* (Fig. 6), indicating that intron acquisition represents a general feature of retained HGT genes in this species. The extent of intron gain observed in *Cuscuta* HGT genes is comparable to that reported following the horizontal transfer of a bacterial amylase gene into Basidiomycetes (Da Lage et al. 2013), suggesting that intron acquisition may be a common outcome of HGT into intron-rich eukaryotic genomes. To our knowledge, this study provides the first evidence among parasitic plants of extensive intron gain following HGT between parasitic plants and their hosts. Among the 27 HGT genes with increased intron numbers, intron gain occurred within protein-coding exons in 9 cases, including *CYP81Q*, whereas the remaining 18 genes exhibited intron gain within untranslated regions. The retention of introns within protein-coding exons suggests that these insertions were subject to selective constraints, as insertions disrupting coding sequences would be expected to impair protein function. In contrast, introns gained within untranslated regions are likely to be more readily tolerated, although they may still influence transcriptional efficiency or mRNA processing.

The expanded introns of *CYP81Q* genes in *Cuscuta* species were frequently colonized by TEs, particularly the nonautonomous DNA transposon *Sto* elements (Hoshino et al. 2016b). The heterogeneous distribution of *Sto* elements among *CYP81Q* loci in different *Cuscuta* lineages following the pHGT event indicates that TE insertions occurred repeatedly and independently after the initial HGT event. Although direct causality cannot be established, the repeated co-occurrence of TE insertions and intron gain across independent *CYP81Q* loci strongly suggests a mechanistic link. This lineage-specific restructuring contrasts with the conserved intron architecture of Lamiales *CYP81Q* genes, which retain a single short intron (Fig. 2).

Interestingly, *hAT* is present only in the first intron of *CcamCYP81Q111* on the BI subgenome, which is closely related to the genome of *C. australis*, but not in that of *CcamCYP81Q110* on the BII subgenome of the allopolyploid *C. campestris* (Fig. 2, Figure S5, Segawa et al. 2026). Considering that the position of *hAT* is similar in both *CcamCYP81Q111* and *CausCYP81Q111* genes, the *hAT* transposition likely occurred once in the common ancestral species with the BI subgenome. In contrast, there is a *Sto* in the first intron of *CcamCYP81Q110* on the BII subgenome instead of *hAT*. This difference in TE composition among *CYP81Q* genes in *Cuscuta* genomes, even within the same subgenus *Grammica*, indicates that these transpositions occurred relatively recently, at least after approximately 3.13 Mya, which is estimated as the divergent time between BI and BII subgenomes in subgenus *Grammica* (Figure S5). However, another possibility, namely that the transposition occurred at the *CYP81Q* gene in the common ancestral genome and subsequently disappeared through transposition in several descendant genomes cannot be excluded. Regardless of which scenario is more likely, both support that these transposons are active in the *Cuscuta* genomes.

DNA transposons termed *Introners* have been shown to generate introns in a wide range of eukaryotes (Huff et al. 2016; Gozashti et al. 2022; Roy et al. 2023; Gozashti et al. 2025), raising the possibility that *Sto* elements may play a related role in intron formation within *Cuscuta* HGT genes. However, unlike characterized *Introners*, *Sto* elements lack experimentally demonstrated splicing motifs capable of directly mediating intron recognition (Huff et al. 2016; Gozashti et al. 2022). The functional consequences of intron gain remain actively debated. Although intron acquisition can increase the risk of splicing errors and associated mutations (Li et al. 2009), introns may also facilitate the integration of foreign genes by enhancing compatibility with the spliceosomal machinery of intron-rich eukaryotic genomes (Da Lage et al. 2013; Takahashi Ueda 2024). In addition, intron expansion may provide a genomic context for the evolution of regulatory 496-features, including intronic noncoding RNAs (Neil and Fairbrother 2019). Although the adaptive significance of intron gain in *Cuscuta* HGT genes remains unresolved, the preferential accumulation of introns accompanied by TE colonization suggests that structural remodeling is an integral component of the long-term retention and functional integration of horizontally transferred genes in parasitic plants.

The functional relevance of HGT-derived genes in recipient plants remains poorly understood, despite numerous studies suggesting the occurrence of HGT in parasitic plant lineages (Zhang et al. 2014; Yang et al. 2016, 2019; Mariault et al. 2025). In this study, the functional relevance of an HGT-derived gene is supported by heterologous expression assays. *Cuscuta* CYP81Q proteins exhibited PSS activity when expressed in yeast, demonstrating that the transferred genes retain full catalytic competence (Fig. 5). These results indicate that *CYP81Q* has been functionally incorporated into the metabolic machinery of *Cuscuta*. Retention of enzymatic activity despite extensive intron expansion and transposon insertions, suggests that the horizontally transferred *CYP81Q* gene is subject to functional constraint, reflecting the biological relevance of sesamin and its related specialized metabolites produced via the *CYP81Q*-dependent pathway in the parasitic lifestyle. Although the physiological functions of sesamin and related lignans in *Cuscuta* remain to be fully resolved, one potential role may involve enhancing seed durability through antioxidative activity during long-distance transoceanic dispersal, as previously proposed (García et al. 2014; Wan et al. 2015). More broadly, these findings demonstrate that pHGT can introduce catalytically functional metabolic enzymes into parasitic plants, raising the possibility that horizontally acquired metabolic capacities contribute to the diversification of specialized metabolic pathways and to the emergence of novel physiological and ecological traits in *Cuscuta*.

### Significance of retained HGT genes in shaping the lignan metabolism of *Cuscuta*

Lignans such as arctigenin and matairesinol, which are synthesized from pinoresinol by PLR, are present in *Ipomoea* species (Lima and Braz-Filho 1997). PLR competes with PSS for the shared substrate, pinoresinol, catalyzing its reduction to secoisolariciresinol rather than its oxidative conversion toward sesamin (Fig. 7a). The occurrence of these lignans in *Ipomoea* therefore implies the presence of functional PLR activity in this lineage. In contrast, no PLR homologs were detected in *Cuscuta* species (Fig. 7b and c), suggesting that ancestral *Cuscuta* lineages lacked PLR genes prior to diversification of the subgenus *Cuscuta*. Given the well-documented metabolic competition between PLR and PSS for pinoresinol (Kim et al. 2009; Murata et al. 2015), the absence of PLR in dodders likely reduced metabolic constraint on the sesamin biosynthetic pathway following horizontal acquisition of PSS. Under these conditions, the introduction of *CYP81Q* via pHGT would be expected to promote sesamin biosynthesis without interfering with pre-existing lignan pathways. Together, these observations indicate that the selective retention of *CYP81Q* genes following HGT contributed directly to the establishment of a functional sesamin biosynthetic capacity in *Cuscuta*. This case illustrates how pHGT can facilitate metabolic innovation in parasitic plants by introducing new enzymatic functions into permissive metabolic contexts, thereby reshaping pathway architecture within existing physiological constraints.

## Materials and methods

### Plant materials

*Cuscuta campestris* seeds were germinated, and seedlings were parasitized onto *N. tabacum* as described previously (Hozumi et al. 2017). Seeds of *Sesamum indicum* cv. “Masekin’ were germinated and grown in potting soil (Sukoyakabaido, Yanmar, Osaka, Japan) mixed with the same volume of vermiculite (GS30L, Nittai Co., Ltd., Aichi, Japan) under natural sunlight from May to August in 2019 in the laboratory (Osaka Metropolitan University, Japan). Mature stems of 4- to 6-week-old *S. indicum* were then parasitized by *C. campestris*. Stems of *S. indicum* and *C. campestris*, the parasitic interface, and *S. indicum* leaves were harvested at 6 days following parasitization, frozen in liquid nitrogen, and stored at −80 °C until analysis. Mechanical wounding of the *S. indicum* stem was performed with a razor blade, and stems within 1.5 cm of the incision were harvested. Stems of *C. campestris*, measuring 10.5–13.5 cm in length and located at the parasitic interface, were harvested, washed twice with 70% ethanol for 2 min, and rinsed with nuclease-free water for 2 min to clean the tissue surface. *Cuscuta epithymum* and *C. europaea* were provided by Dr. J. Macas (Biology Centre, Czech Academy of Sciences). Specimens of naturally occurring *Cuscuta chinensis* and *Artemisia indica* were harvested in Tokushima Prefecture and Shiga Prefecture, Japan, respectively.

### Chemicals

(+)-Sesamin (ChromaDex, Los Angeles, CA), (+)-sesaminol (Nagara Science Co., Ltd., Gifu, Japan), (+)-pinoresinol (Sigma-Aldrich Co. LLC, St. Louis, MO), and previously synthesized (+)-piperitol (Noguchi et al. 2014), were prepared for use as the reference samples.

### LC-MS analysis

Lignans in extracts of flowers and seeds of *Cuscuta* plants (*C. campestris*, *C. europaea*, *C. epithymum*, *C. chinensis*, and *C. japonica*) were analyzed as described previously (Murata et al. 2017; Ono et al. 2018). A 10 mg sample of lyophilized flowers or seeds was homogenized to a fine powder using a TissueLyser II (Qiagen, Hilden, Germany). Next, 1 mL of 70% acetonitrile aqueous solution was added to the homogenized sample, followed by ultrasonic extraction at room temperature for 2 min. The resulting extracts were filtered and analyzed using an ion-trap time-of-flight mass spectrometer (LCMS-IT-TOF, Shimadzu Corp., Kyoto, Japan) equipped with a photodiode array detector (Shimadzu Corp.). Chromatographic separation was performed using a C18 column (TA12S03-1503WT, YMC Co., Ltd., Kyoto, Japan); 150 mm × 3 mm i.d., 3 µm particle size). The mobile phases were A, 0.1% HCO_2_H-H_2_O and B, 0.1% HCO_2_H-MeOH. A linear gradient of B was applied as follows: 30–50–90–90–30–30% B at 0–10–25–32–32.01–40 min, at a flow rate of 0.3 mL/min.

### PCR and reverse transcription-PCR (RT-PCR)

Genomic DNA was extracted from stem tissue using a DNeasy Plant Mini kit (Qiagen), according to the manufacturer's instructions. Total RNA was isolated using a RNeasy Plant Mini kit (Qiagen), according to the manufacturer's instructions. The RNA samples were treated with either a DNase Set (Qiagen) or a TURBO DNA-free Kit (Thermo Fisher Scientific, Waltham, MA). After DNase treatment, cDNA was synthesized using an oligo dT primer and a PrimeScript RT Reagent Kit (Takara Bio Inc., Kusatsu, Japan), according to the manufacturer's instructions. PCR products were amplified using gene-specific primer sets (Table S9), as described previously (Ono et al. 2018).

### Genome and transcriptome

Publicly available *Cuscuta* transcriptome and genome datasets were used in this study. RNA-seq data for *C. campestris* were obtained from the DNA Data Bank of Japan Sequenced Read Archive (Accession number DRX195090; https://www.ncbi.nlm.nih.gov/sra/DRX195090) (Kaga et al. 2020). The assembled genome sequence and annotations for *C. campestris* were obtained from the plaBi database (http://plabipd.de/portal/cuscuta-campestris) and for *C. australis* from the National Center for Biotechnology Information (NCBI) Sequence Read Archive (https://www.ncbi.nlm.nih.gov/bioproject/PRJNA394036). *CcCPR1*, *CcCYP81Q110*, *CcCYP81Q111*, *CcCYP81AX6*, and *CaCYP81Q111* correspond to contigs Cc043955.t1, Cc046292.t1, Cc015414.t1, Cc047366.t1, and RAL50776 (C65N0022E0.1), respectively. DNA-seq data for *C. americana* were obtained from the Sequence Read Archive (SRA) experiment ERR3569498 of BioProject PRJEB34450, for *C. californica* were obtained from ERR3569499, and for *C. gronovii* were obtained from ERR3569502. Synteny analysis was performed using BLASTP to identify the best-hit protein sequences.

### Molecular phylogenetic analysis

Genes from the genus *Cuscuta* that are similar to the *SiCYP81Q1* were identified using BLASTX. The search used an E-value cutoff of 1e-180 and a bit score cutoff of 500 against protein data from the assemblies of GCA_003260385.1, GCA_900332095.2, GCA_945859875.1, and GCA_945859915.1. Genes from *C. australis, C. campestris, C. europaea,* and *C. epithymum* that met these criteria were then used as queries in a BLASTX search against the nr database, applying an E-value cutoff of 1e-100. Subsequently, genes from the genus *Cuscuta* and the order Lamiales were manually selected. Genes from *Ipomoea nil* and *Glycine max* that are similar to the *SiCYP81Q1* cDNA were identified through separate BLASTX against these species. The deduced amino acid sequences of the *Cuscuta CYP81Q* genes and *CYP81-*related genes used in this study are shown in Table S4. The phylogenetic tree shown in Fig. 3 was constructed by aligning the entire length of the proteins using the maximum likelihood (ML) method implemented in *SeaView* with PhyML (ln(L)=−23653.0; 1868 sites; GTR model with 4 rate categories) (Gouy et al. 2010). The phylogenetic tree of exon 2 was constructed using the BioNJ method in *SeaView* with 373 aligned sites and 1000 bootstrap replications (Gouy et al. 2010). The estimated divergence times of *Cuscuta* species were inferred as in Figure S5 using the method described in Method. Synteny analysis was performed based on BLASTP search results (cutoff, 1.00e-30).

### Genome comparisons

Genomic sequences spanning 5 kb regions in both the upstream and downstream directions of *CYP81Q* genes were obtained based on the nucleotide sequences shown in the Nucleotide ID of Table S5. Pairwise similarities between these sequences were analyzed using *promer* implemented in the *MUMmer* -v3.1.0 package (Kurtz et al. 2004). Structural similarities were visualized with *dotplots* using *mummerplot*.

### Molecular cloning

The nucleotide sequences of coding regions of the *Cuscuta CYP81*-related genes used in this study are shown in Table S8. cDNAs of the *CYP81Q* genes and *CYP81-*related genes were obtained by RT-PCR from total RNA using gene-specific primers shown in Table S9. *CausCYP81Q111* was artificially synthetized without codon-optimization (Eurofins Genomics, Luxembourg City, Luxembourg), based on the sequence of RAL50776. All P450 genes were cloned into yeast expression vectors and heterologously coexpressed in yeast with *C. campestris* cytochrome P450 reductase, *CcCPR1* (Cc043955.t1), as described previously (Murata et al. 2017).

### Biochemical analysis

Biochemical analysis of *Cuscuta* CYP81Q proteins was performed as described previously with minor modifications (Murata et al. 2017). Briefly, yeast cells expressing *Cuscuta CYP* genes were precultured overnight at 30 °C with rotary shaking at 120 rpm in 3 mL of synthetic defined liquid medium supplemented with the appropriate amino acids for each expression vector. Stationary-phase cultures (50 mL) were transferred into 1 mL of fresh medium in 24-well plates, supplemented with 100 µM of lignan substrates. The cultures were incubated for a further 24 h at 30 °C with rotary shaking at 120 rpm. For extraction, cells were harvested together with the medium and disrupted by sonication. The homogenate (50 µL) was mixed with 50 µL acetonitrile and centrifuged at 21,000×g for 10 min. The supernatant was collected, filtered through a Millex-LH syringe filter (Merck Millipore, Burlington, MA), and subjected to HPLC analysis. Briefly, the filtered reaction products were separated using a Cortecs UPLC C18+ column (part# 186007401, 2.7 µm, 3 mm × 75 mm, Waters Corporation, Milford, MA). Chromatographic separation was performed with mobile phases A (0.1% trifluoroacetic acid-H2O) and B (0.1% trifluoroacetic acid-acetonitrile), using a linear gradient of 30–80–80–30–30% B at 0–1.4–1.8–2.0–2.5 min, at a flow rate of 1.25 mL/min. Lignan products were detected using a photodiode array detector at 280 nm.

### Preparation and imaging of agarose-embedded sections

Parasitic interface tissues were fixed in 4% (w/v) paraformaldehyde in phosphate-buffered solution (Fujifilm Wako Pure Chemical Corporation, Osaka, Japan), embedded in 8% (w/v) agarose, and sectioned into 200-μm sections using a MicroSlicer^TM^ ZERO 1N (DOSAKA, Kyoto, Japan). Histochemical staining of sections was performed using a 0.5% (w/v) aqueous solution of Toluidine Blue O (1B-481, Waldeck GmbH & Co., Munster, Germany). Stained sections were observed using a BX53 Biological Microscope (Olympus, Tokyo, Japan).

### Identification of TEs

TEs and their footprints within the intronic regions of *CYP81Q* genes were identified and annotated through manual curation. To search for insertions and deletions and to visualize internal structural features within the expanded introns, we utilized dot-plot analysis using the Harrplot program implemented in the GENETYX software (Nihon Server., Tokyo, Japan), with a window size of 10 bp and a minimum match requirement of 8 bp. To identify repetitive sequences, sequence similarities were evaluated using BLASTN searches against the Whole Genome Shotgun database (limited to the genus *Cuscuta*) via the NCBI website. Based on these combined results, the exact boundaries and classification of the inserted TEs were determined by manually searching for characteristic hallmarks, including terminal inverted repeats and target site duplications. Finally, multiple sequence alignments were generated using CLUSTALW on the GenomeNet server to further compare and verify the identified sequences.

### Analysis of the number of introns per gene

A list of HGT genes in *C. campestris* and their putative species was obtained from previous literature (Vogel et al. 2018). The number of introns per gene was determined from gene feature files associated with GenBank accession numbers listed in Table S7. The highest-similarity pairs of non-HGT genes were identified using BLASTP, with all other genes not included in the HGT gene list serving as queries. Differences in intron number per gene, determined from gene feature files, were assessed using a random sample of 50 gene pairs, and the average frequencies were calculated using 2000 iterations of random sampling.

### Gene synteny and phylogenetic analyses of PLR-related genes

To identify homologs of *Forsythia* × *intermedia* PLR (AAC49608.1), DIAMOND BLASTP v2.1.12 (Buchfink et al. 2021) was used to search predicted amino acid sequences from *C. campestris* (t267557.G001, Plant GARDEN; https://plantgarden.jp/en/download/), *C. chinensis* (t132261, Plant GARDEN), *C. europaea* (GCA_945859875.1), *I. nil* (Asagao_chr_1.2, Morning Glory Genome Consortium; http://viewer.shigen.info/asagao/index.php), *I. triloba* (GCF_003576645.1), *S. lycopersicum* (GCF_000188115.5), and *A. thaliana* (GCA_000001735.1). Identified homologous sequences were aligned using MAFFT v7.525 (−maxiterate 1000 –localpair) (Katoh et al. 2005), and phylogenetic reconstruction was performed using IQ-TREE v2.3.6 (-m MFP -bb 1000 -alrt 1000) (Minh et al. 2020). For synteny analysis, all genes located on the contigs harboring the PLR homolog most closely related to *F.* *×* *intermedia* were subjected to homology search using DIAMOND BLASTP. Syntenic blocks were detected with MCScanX (Wang et al. 2012) and visualized with SynVisio (Bandi and Gutwin 2020).

## Supplementary Material

kiag335_Supplementary_Data

## Data Availability

Data supporting the findings of this study are available in Figures S1–S11, and Tables S1–S9. The sequencing data are available from the DNA Data Bank of Japan under accession numbers PRJDB13351 and PRJDB15151.

## Preprint server

This manuscript has been posted to *bioRxiv*, with the DOI: https://doi.org/10.1101/2025.08.25.672084.
