# Supplementary material for: Transposon-colonized intron gain follows parasitism-mediated horizontal transfer of a cytochrome P450 gene
Source: Plant Physiol. 2026 Jun 30;201(2):kiag335. doi: 10.1093/plphys/kiag335 (PMC13591195; doi:10.1093/plphys/kiag335)
Supplement: Supplementary file 1 [file kiag335_Supplementary_Data.zip › PLPHYS-2026-0303R1_Suppl Figs_2.pdf]

Supplementary materials for

Transposon-colonized intron gain follows parasitism-mediated horizontal transfer of a cytochrome P450 gene

Eiichiro Ono[1,#,*], Kohki Shimizu[2,#], Jun Murata[3], Akira Shiraishi[3], Ryusuke Yokoyama[4], Hiromi Toyonaga[1], Tenta Segawa[1], Masaki Takagawa[4], Manabu Horikawa[3], Atsushi Hoshino[5,6], Koh Aoki[2,7,*]

[1]Research Institute, Suntory Global Innovation Center (SIC) Ltd., 8-1-1 Seikadai, Seika-cho, Soraku-gun, Kyoto 619-0284, Japan
[2]Graduate School of Life and Environmental Sciences, Osaka Prefecture University, 1-1 Gakuen-cho, Naka-ku, Sakai, Osaka 599-8531, Japan
[3]Bioorganic Research Institute, Suntory Foundation for Life Sciences (SUNBOR), 8-1-1 Seikadai, Seika-cho, Soraku-gun, Kyoto 619-0284, Japan
[4]Evolutionary Genomics, Graduate School of Life Science, Tohoku University, 6-3 Aramaki Aza-Aoba, Aoba-ku, Sendai, Miyagi 980-8578, Japan
[5]National Institute for Basic Biology (NIBB), 38 Nishigonaka, Myodaiji, Okazaki, Aichi 444-8585, Japan
[6]Graduate Institute for Advanced Studies, SOKENDAI (The Graduate University for Advanced Studies), 38 Nishigonaka, Myodaiji, Okazaki, Aichi 444-8585, Japan
[7]Graduate School of Agriculture, Osaka Metropolitan University, 1-1 Gakuen-cho, Naka-ku, Sakai, Osaka 599-8531, Japan

Email: Eiichiro_Ono@suntory.co.jp, kaoki@plant.osakafu-u.ac.jp

This PDF file includes:

Figures S1 to S11
Supplementary Methods
Supplementary References

Other supporting materials for this manuscript include the following:

Tables S1 to S9

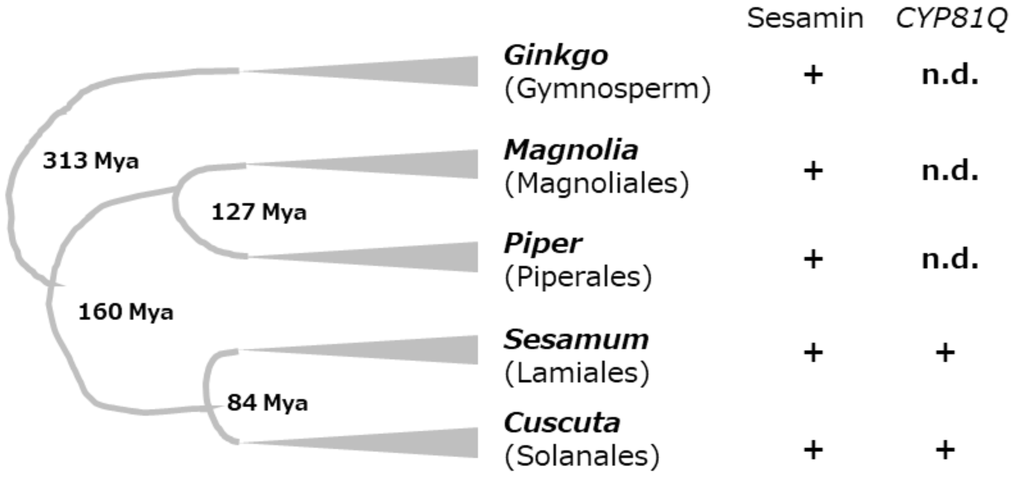

Fig. S1. Distribution of sesamin and *CYP81Q* gene in plant kingdom

Sesamin-producing plants are present sporadically in the plant phylogeny. Numbers on the tree indicate estimated divergent times according to the *Timetree* time scale of life28. "+" indicates detected. n.d. indicates not detected in a BLAST search querying *SiCYP81Q1* genes with default settings versus public genomes of *Ginkgo biloba* (GCA_024626585.1), *Magnolia ashei* (GCA_003571905.1), and *Piper nigrum* (Accession number PRJNA529758). Mya, million years ago.

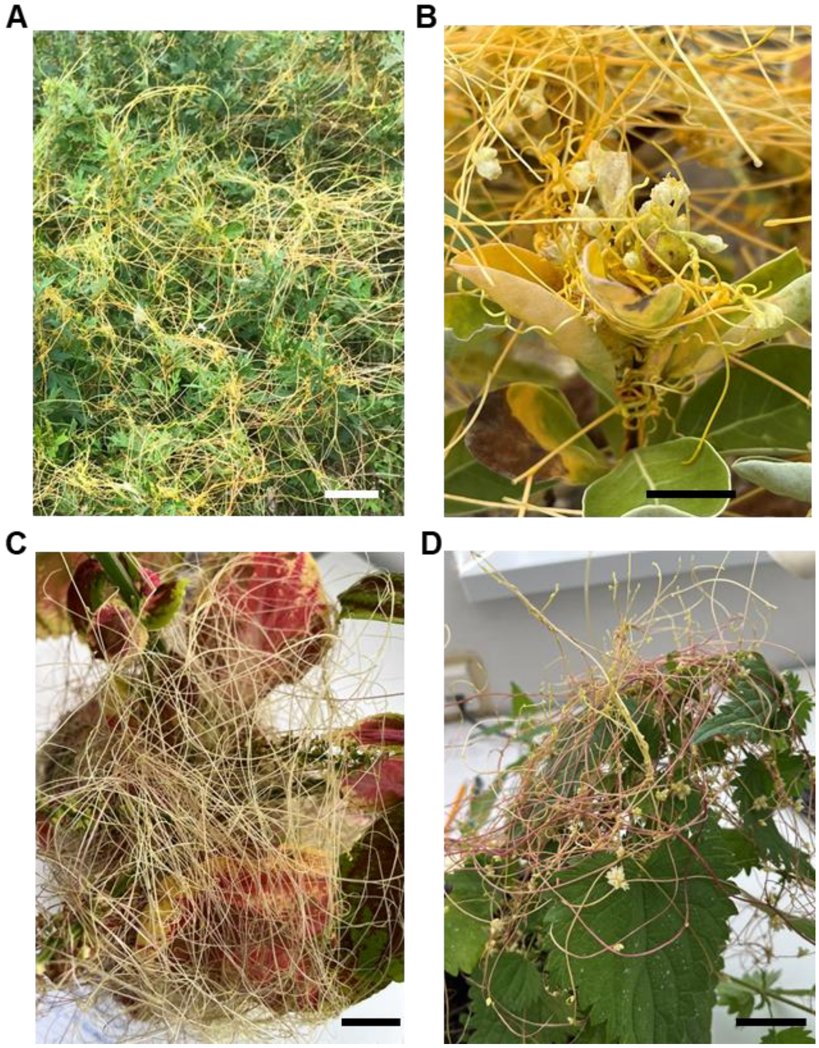

Fig. S2. Appearance of *Cuscuta* species used in this study.

A) *Cuscuta campestris* parasitizing host plants (*Artemisia indica*) along the coast of Lake Biwa, Shiga Prefecture, Japan. White scale bar: 10 cm. **B)** *C. chinensis* parasitizing *Vitex* spp **C)** *C. epithymum* parasitizing *Coleus* spp. **D)** *C. europaea* parasitizing *Urtica* spp. Black size bar: 5 cm.

A

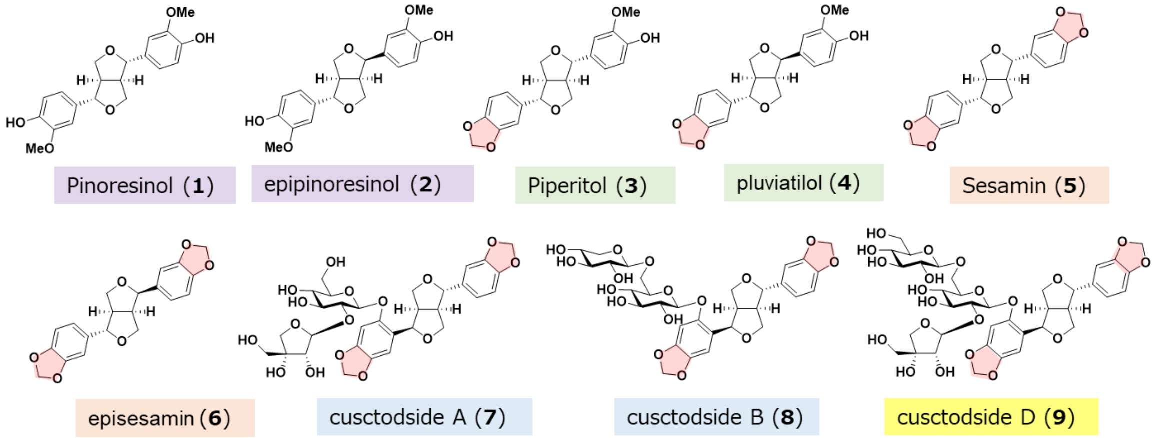

B

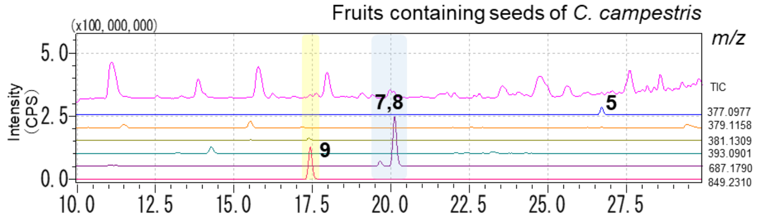

Fig. S3. Lignans in *Cuscuta* plants

A) Lignans detected in *Cuscuta* species. Lignans: (1) pinoresinol, (2) epipinoresinol, (3) piperitol, (5) sesamin, (7) cuscutoside A, (8) cuscutoside B, and (9) cuscutoside D (Yahara et al. 1994, Xiang et al. 2001). Lignans were detected using liquid chromatography-mass spectrometry (LC-MS) analysis. Authentic standards, (4) pluviatilol and (6) episesamin, were analyzed; however, neither peak was detected in *Cuscuta* species. **B)** Cuscutoside A (7), cuscutoside B (8), and cuscutoside D (9), in addition to lignans detected in Figure 1, were detected in the seed-containing fruits of *C. campestris*. Assignment of peaks 7 and 8 to the reported structures is tentative, as retention time information is unavailable in previous reports (Yahara et al. 1994).

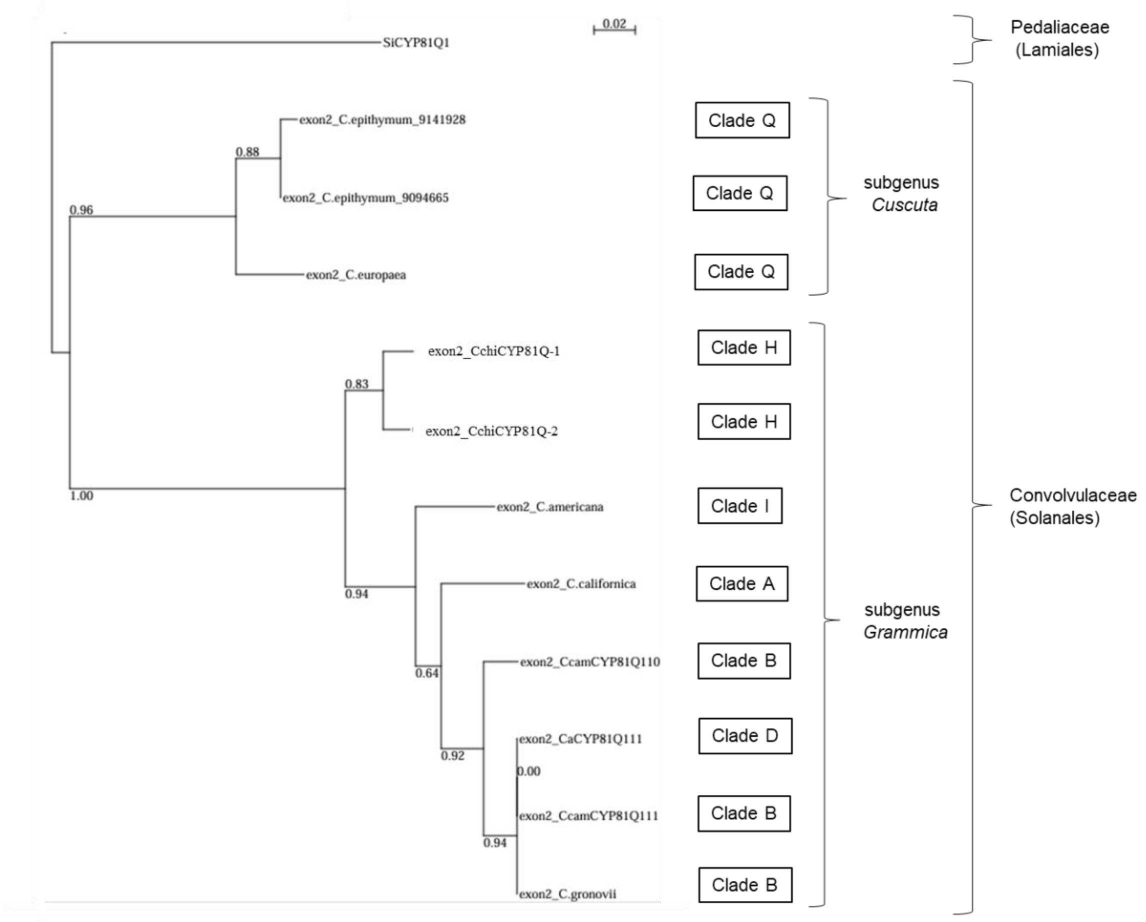

Fig. S4. Phylogeny of exon 2 of *CYP81Q* genes

NGS data (SRA IDs ERR3569498, ERR3569499, SRR2143215, ERR3651373, and ERR3569501) for three *Cuscuta* spp. (*C. americana*, *C. californica*, and *C. gronovii*) were assembled using Trinity ver.2.9.1 (Grabherr et al. 2011). Contigs showing the highest similarity with *CcCYP81Q110* or *CcCYP81Q111* were selected by BLASTN search for each Trinity assembly. A phylogenetic tree was constructed by maximum likelihood using Seaview software (phyML: ln(L)=-1333.8, 387 sites, bootstrap=100 rep., GTR 4 rate classes) (Gouy et al. 2010). The numbers at the branches indicate bootstrap values. The sequence of *SiCYP81Q1* exon 1, which corresponds to exon 2 of *Cuscuta CYP81Q*-related genes, was used as the outgroup. The exon 2 sequences from *C. americana*, *C. californica*, and *C. gronovii* that belongs to sugenus *Grammica* were co-clustered with those of *C. chinensis*, *C. australis* and *C. campestris*. The two sequences from subgenus *Cuscuta*, *C. europaea* and *C. epithymum*, formed a distinct clade from that of *Grammica*, roughly in line with the phylogeny of *Cuscuta* spp (García et al. 2014). The exon 2 nucleotide sequences for *CYP81Q*-related genes are available in *Supporting Information* Data S1. Phylogenetic clade (B to Q) is labeled with species names as defined in a previous phylogenetic study (García et al. 2014).

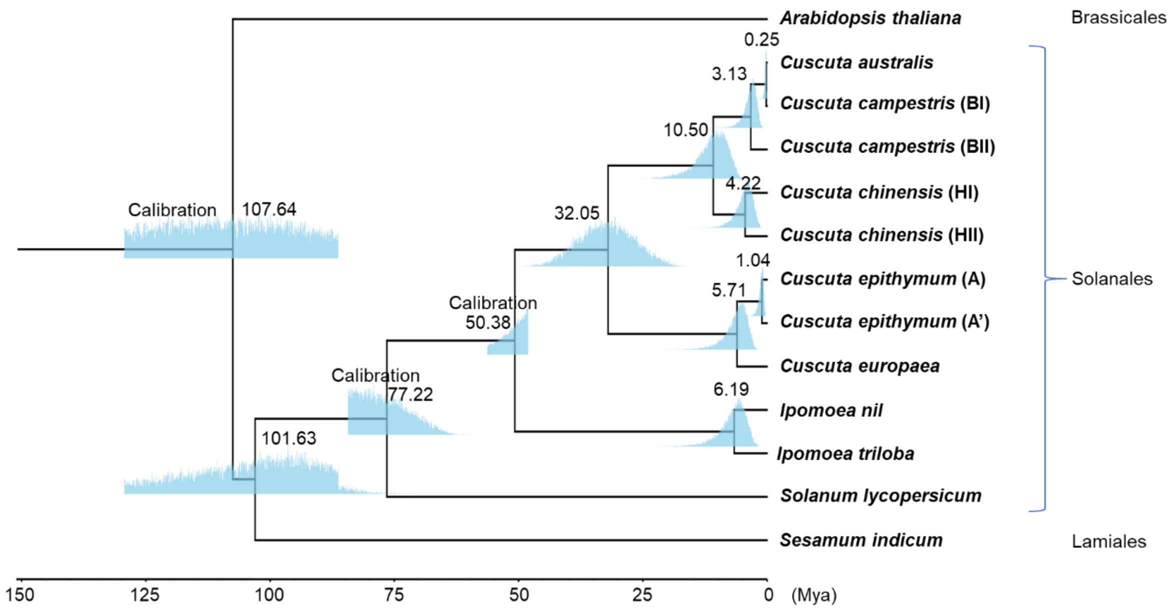

Fig. S5. Estimated divergent time of *Cuscuta* species

The blue shaded regions indicate the posterior distributions of node ages inferred from a Markov chain of 20,000,000 iterations. The first 20% of the samples were discarded as burn-in, resulting in 16,000,000 effective samples used for estimation. Median million years ago (Mya) values are shown at each node. Detailed method for the estimation of divergence time is described in the Supplementary Method.

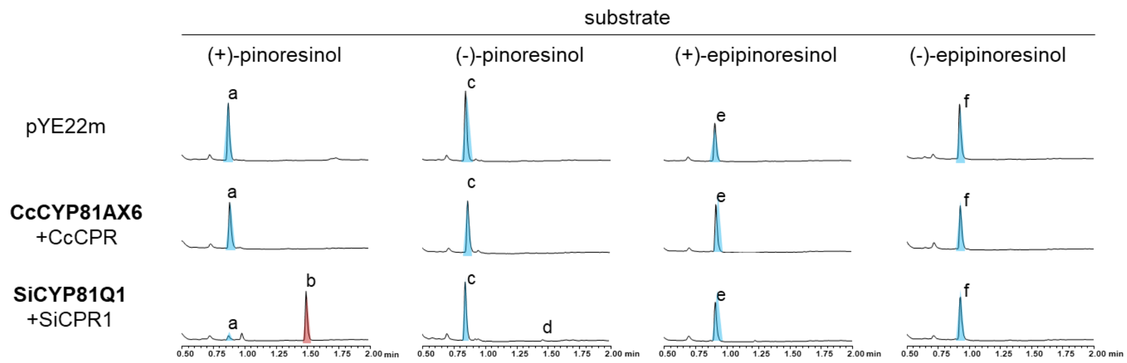

Fig. S6. Enzymatic activity of CcCYP81AX6

(Top) Negative control (empty yeast expression vector, pYE22m) for the bioconversion assay. Only the tested substrate (blue) was observed. (Middle) No product was observed in assays in which CcCYP81AX6 was co-expressed with CcCPR1. (Bottom) In reference assays in which SiCYP81Q1 was co-expressed with SiCPR1, (+)-pinoresinol was converted to (+)-sesamin (red), as shown in a previous report[14]. These results indicate that CcCYP81AX6 has no PSS activity. a, (+)-pinoresinol; b, (+)-sesamin; c, (-)-pinoresinol; d, (-)-sesamin; e, (+)-epipinoresinol; f, (-)-epipinoresinol.

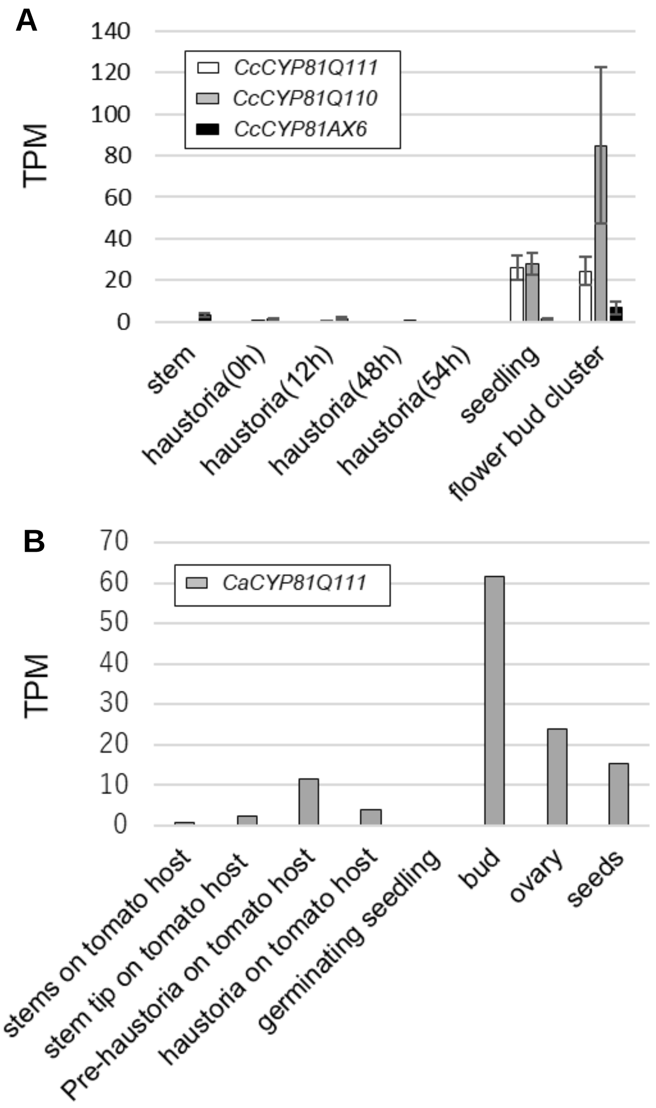

Fig. S7. Expression of *Cuscuta CYP81Q* genes in various tissues

A) RNA-seq analyses of *CYP81Q*-related genes in *Cuscuta campestris*. Each bar indicates the mean and standard deviation for three replicates. TPM values were estimated based on mapping of RNA-seq reads in DRA009453 (Kaga et al. 2020) onto cucam_0.32.annot.cds.fasta from the plaBi database (http://plabipd.de/portal/cuscuta-campestris) (Vogel et al. 2018). **B)** RNA-seq analyses of *CYP81Q*-related genes in *Cuscuta australis*. Each bar indicates the estimated TPM from a single-replicate RNA-seq experiment in PRJNA394036 (Sun et al. 2018). TPM, transcripts per million.

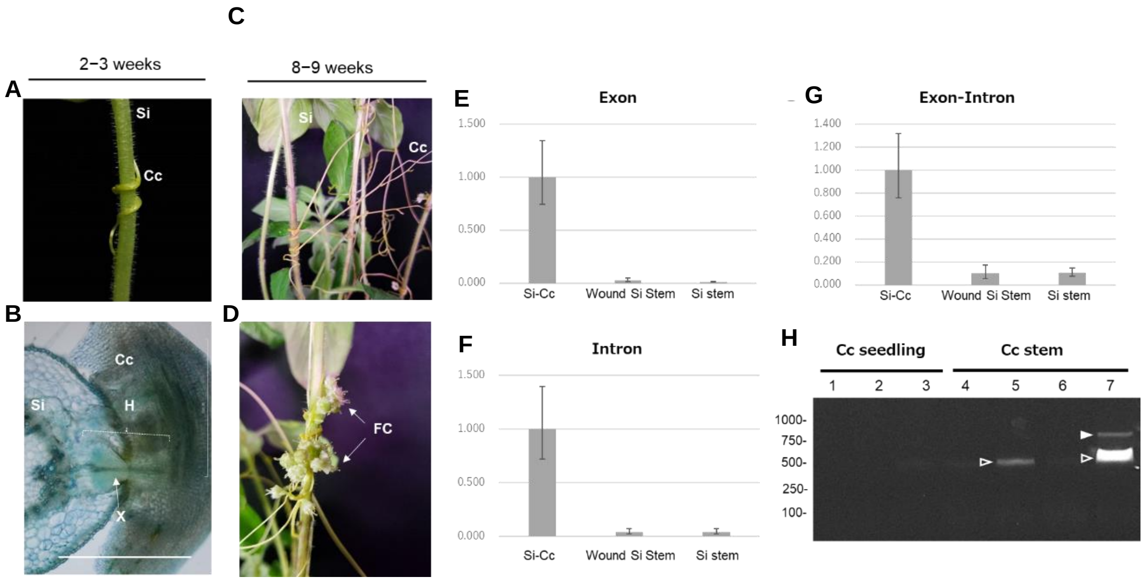

Fig. S8. Parasitism of *Cuscuta campestris* on *Sesamum indicum* and transfer of *SiCYP81Q1* mRNA

A) Parasitism of the parasite, *Cuscuta campestris* (Cc), on the host, *Sesamum indicum* cv. Masekin (Si), at 2-3 weeks after germination. **B)** Cross section (thickness=200 μm) of haustorium with toluidine blue staining at 2-3 weeks after germination. Cc, *C. campestris*; Si, *S. indicum*; H, haustorium; X, haustorial xylem. Scale bar, 500 μm. **C)** *C. campestris* (Cc) on *S. indicum* cv. Masekin (Si) at 8-9 weeks after germination. **D)** *C. campestris* formed a flower and developed fertile seeds. FC, floral cluster of the parasite. Gene expression of *Si_CYP81Q1* (**E)** exon, **F)** intron, **G)** exon-intron) in stems of *S. indicum* at the parasitic interface of *C. campestris* (left), at mechanical wounded site (middle) and non-treatment (right). **H)** RT-PCR detection of *Si_CYP81Q1* in the non-parasitizing stems (Cc seedlings) and parasitizing stems of *C. campestris* (Cc stem). Each lane indicates an independent replicate. The spliced mRNAs were detected in lanes 5 and 7 (lower bands, open arrowheads) and an intron-retained mRNA was detected in lane 7 (upper band, closed arrowhead).

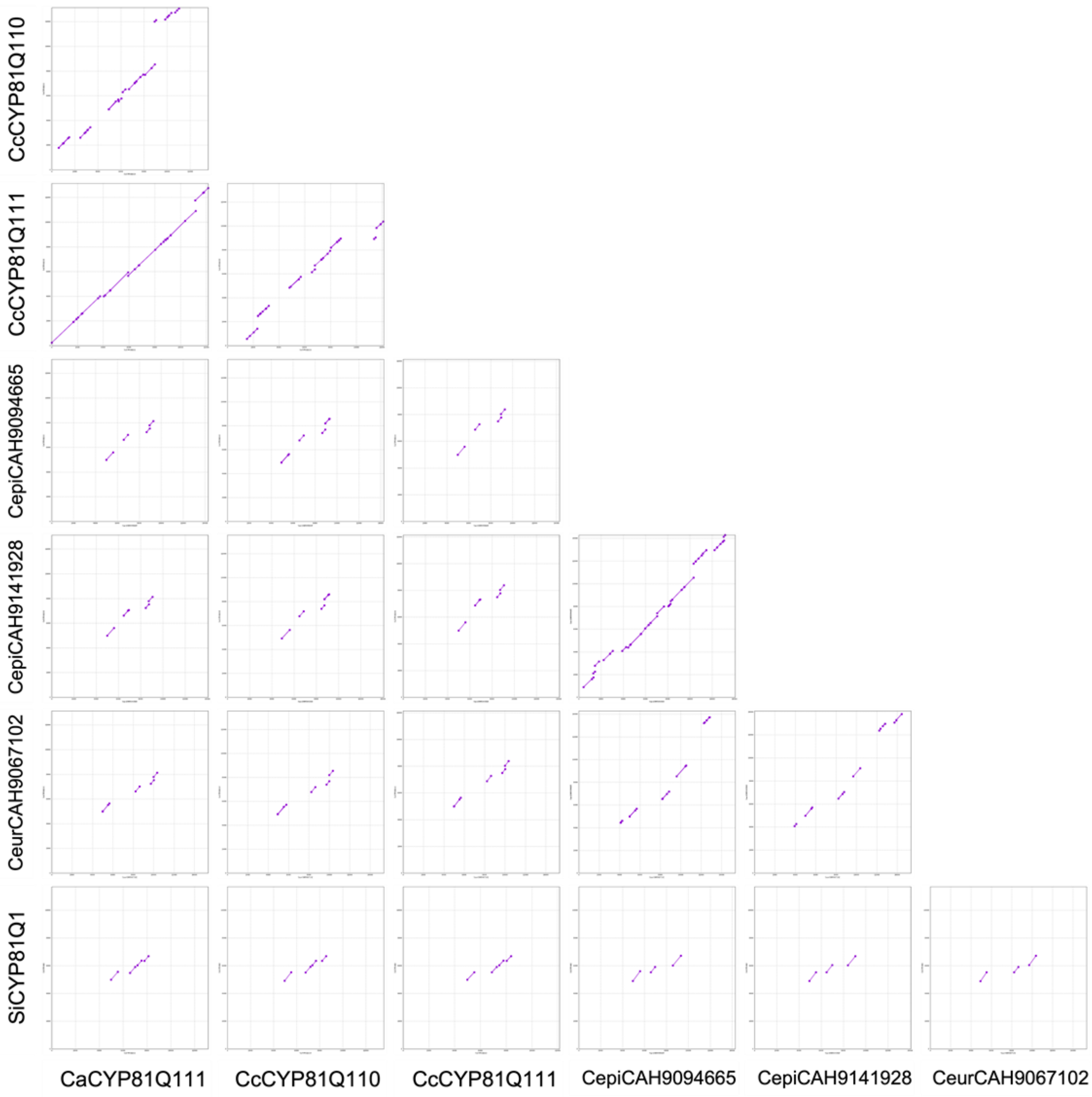

Fig. S9. Genome-by-genome alignment of genomic regions containing *CYP81Q* genes (plus/minus 5kbp)

Structural similarity in a broad genomic region (~10 kb with *CYP81Q* in the center) was evaluated using a dot-plotting tools. Alignment of *Cuscuta* genomic sequences performed using promer in the Mummer -v3.1.0 package (Kurtz et al. 2004). The plots show structural similarity of *C. campestris*, *C. australis*, *C. epithymum*, *C. europaea* and *S. indicum*. Purple dots indicate unique forward alignments. Structural similarities were visualized with dot plots using mummerplot. Only exons share structural similarity. These results are consistent with the idea that either 1) non-coding regions outside of *PSS* genes change easily due to loosened constraints for nucleic acid alteration as compared to the coding region, or 2) a small genomic fragment including the *PSS* ORF was transferred in a parasitism-mediated horizontal gene transfer event.

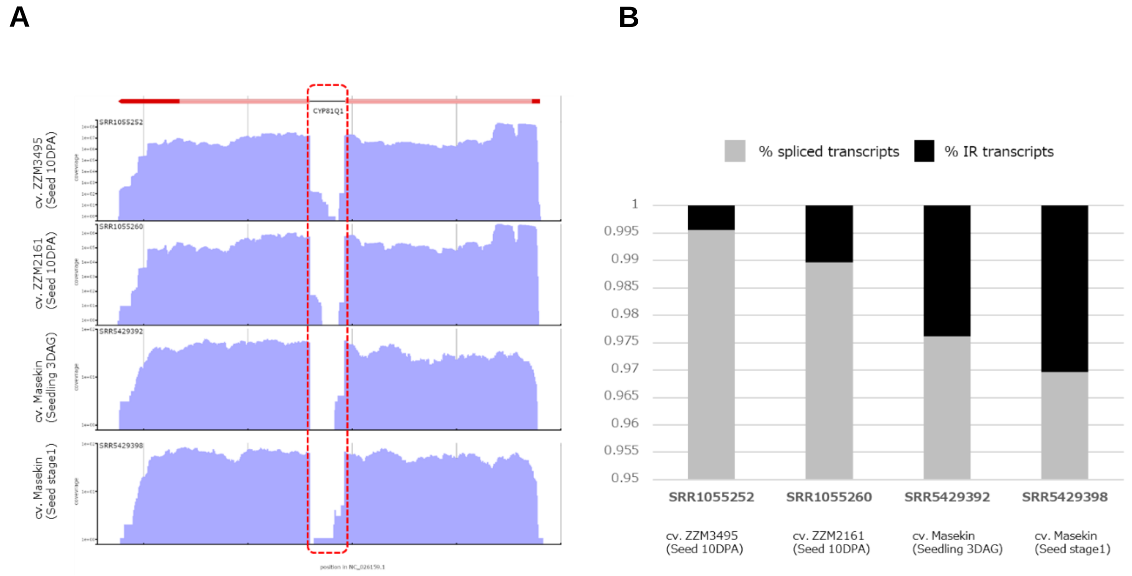

Fig. S10. Investigation of intron retention (IR) transcripts of *SiCYP81Q1* in sesame seeds

A) RNA-seq data from *Sesamum indicum* seeds of four cultivars (SRR1055252, SR1055260, SRR5429392, and SRR5429398) were mapped on the *S. indicum* genome using the *HISAT2* aligner (Kim et al. 2019). Identification of a small portion of RNA reads covering the intron of the *CYP81Q1* gene (NP_001306620), as indicated by the dotted red box, supports the idea that IR-transcripts of *CYP81Q1* gene are expressed as minor transcripts. **B)** Reads covering exon-intron junctions and reads in the intron are minor transcripts, accounting for < 3% of *CYP81Q1* transcripts. We counted numbers of reads (1) covering exon 1-exon 2, (2) mapping to exons, (3) covering exon 1-intron, (4) covering intron-exon 2, and (5) mapping to the intron, and then calculated splicing efficiency (6) using the following formula: (2)/{(2)+[(3)+(4)]}/2. Intron retention (7) was estimated as 1-(6). Relative abundance of spliced transcripts (6), black; of IR-transcripts (7), gray (graph on the right). These results suggest the presence of a very small portion of *IR-SiCYP81Q1* transcripts. Seed developmental stage was described in Ono et al. 2006. DAG, days after gemination; DPA, days post anthesis.

A

```
Intron 1
Cc_CYP81Q110    17   GTACTTT--GTA Cc_Sto1 TATTTCTTTT 284
Cc_CYP81Q111    17   GTACTATACATA TG      TATTTCTTTT  40
Ca_CYP81Q111    17   GTACTATACATA TG      TATTTCTTTT  40

Intron 3
Cc_CYP81Q110    119  TTGATATATA Cc_Sto2 TAGTCCTTTT   406
Cc_CYP81Q111    114  TTGACATATA CGGAG   TAGTGTTTTT   138
Ca_CYP81Q111    114  TTGACATATA CGGAG   TAGTGTTTTT   138
```

B

```
InSto1 5' TIR   CTCCCTCAGTC
InSto1 3' TIR   CTCCATCCGTC
Cc_Sto1 5' TIR  CTCCCTCCGTT
Cc_Sto1 3' TIR  CTCCATCCGTT
Cc_Sto2 5' TIR  CTCCCTCTGTC
Cc_Sto2 3' TIR  CTCCCTCCGTC
```

C

```
Cc_Sto1    CTCCCTCCGTTCTCAAAAATACTTCTTCTTTCATTT---------CCAACAAAACACTTC  51
Cc_Sto2    CTCCCTCTGTCCCAGAAAATCTTTCTCCTTTCTTTTTTCATCTGTCCCAAAAAACATTTC  60
           ******* ** *   *****  **** ***** ***         ** * ****** ***

Cc_Sto1    CACTTTCTATAAAAAA---ATACTTTACCCTTACTTTTTCATACCCTATCTATCACATCA 108
Cc_Sto2    TCATTTCTATTAAAAAGTCACTTTTACCCCTTACTTTTTCATACTCTACCTATCGCATCA 120
              ******* *****   *   **  ***************** *** ***** *****

Cc_Sto1    TATTTTTTCTCTAATAATC----TATCTATCACATCTTACTTTTACCCATCATATCAAGG 164
Cc_Sto2    TACTTTTTTACTAATAATCGATCTATCCATCAAATCATACTTTTACCCATCACATCAAAG 180
           ** *****  *********    **** **** *** *************** ***** *


Cc_Sto1    -ACAAAATTGGAAGCCAACTACAATTTAACACTTCCTCATTTTTACACTTGG---TCAAA 220
Cc_Sto2    GACATTTTTGTAAATTCAATACTAAAATACACTTCCCTTAAATCCCCCTTAAAAAACAAA 240
            ***   *** **    * *** *    ********      *  * ***      ****

Cc_Sto1    ATAAGAACTTTTTTTAGAACGGATGGAG                                 248
Cc_Sto2    ACTTTCATGTTTTCCGGGACGGAGGGAG                                 268
           *     *  ****   * ***** ****
```

Fig. S11. DNA transposons in introns of *Cuscuta CYP81Q* genes

A) Comparison of the intron 1 and 3 sequences. The TA dinucleotide target site duplication is indicated in boldface. The underlined sequences are the putative footprints of the *Stowaway* elements. Numerals indicate sequence positions in the intron sequences. **B)** Comparison of the conserved TIR sequences of *Stowaway* elements. **C)** Comparison of entire sequences of *CcSto1* and *CcSto2*. Terminal inverted repeat (TIR) sequences are underlined.

D

Intron 1

Cc_CYP81Q110	522	AATTGATTGCCACTATGC		TTATTTTTT	549
Cc_CYP81Q111	270	AATTGATTGT**AAGTGAGT**	*Cc_hAT1*	**TAGTCAGT**TTAATTTTTT	687
Ca_CYP81Q111	270	AATTGATTGT**AAGTGAGT**	*Ca_hAT1*	**TAGTCAGT**TTAATTTTTT	719

E

Tip100 5´	TIR	CAGGGGCGGAGGCA
Tip100 3´	TIR	CAGGGGCGGAGCCA
Cc_hAT1 5´	TIR	CAGTGGCGGAACCA
Cc_hAT1 3´	TIR	CAGTGGCGGAGCCA
Ca_hAT1 5´	TIR	CAGTGGCGGAACCA
Ca_hAT1 3´	TIR	CAGTGGCGGAGCCA

F

```
Cc_hAT1    CAGTGGCGGAACCACATGGGAGCAAATGACTACAATTGCAGGTAGTCCCTTTTCTATATA   60
Ca_hAT1    CAGTGGCGGAACCACATGGGAGCAAATGACTACAATTGCAGGTAGTCCCTTTTCTATATA   60
           ************************************************************

Cc_hAT1    TATATATATATATATATAAATAT-TATATATATATATATAT-----------------TA   102
Ca_hAT1    TATATATATATATATATATATATATATATATATATATATATATATATATATATATATATA   120
           ****************** **** *****************                 **

Cc_hAT1    TATATTATATTATAATATATATATATA-------------------ATATATATAGTACA   143
Ca_hAT1    TATATTATATATATATATATATATATATATATATATATATATATATATATATATAGTACA   180
           **********    *************                   **************

Cc_hAT1    AAGAGTACAAGCAAACCTAAAAAAAAGAGTACCGGGGAGGGAAAGAAGCACTACG---AC   200
Ca_hAT1    AAGAGTACAAGCAAACCTAAAAAAAAGAGTACCGGGGAGGGAAAGAG-CACTACGGACGC   239
           **********************************************  *******    *

Cc_hAT1    CGAGAGCAGAAAGAAGAATAGAAATACTCCTTATTTAAGATTACCATCCATGTCTACGAT   260
Ca_hAT1    GGAGAGCAGAAAGAAGAATAGAAATACTCCTTATTTAAGATTACCATCCATGTCTACGAT   299
           ************************************************************

Cc_hAT1    CAGAGTAAGGAGAAACCATTAGACCAATATTTATTTAAATATGTTCATCTCCGGTAACTG   320
Ca_hAT1    CAGAGTAAGGAGAAACCACTAGACCAATATTTATTTAAATATTTTCATCTCCGGTAACTG   359
           ****************** *********************** *****************

Cc_hAT1    CTAGATACGAACTTTTTCTTCTACTTTGCATCCACTGGTGTTGGATCTTGGCTCCGCCAC   380
Ca_hAT1    CTAGATACGAACTTTTTCTTCTACTTTGCAT-------TGTTGGATCTTGGCTCCGCCAC   412
           *******************************       **********************

Cc_hAT1    TG                                                             382
Ca_hAT1    TG                                                             414
           **
```

Fig. S11. DNA transposons in introns of *Cuscuta CYP81Q* genes (continued)

D) Comparison of the intron 1 sequences. Putative 8-bp target site duplication is indicated in boldface. Numerals indicate sequence positions in the intron sequences. Due to the low similarity of the target sequence region between *CcamCYP810Q110* and *CcamCYP810Q111* genes, it is possible that the *hAT* element was past inserted into the *CcamCYP810Q111* gene and exited. If this is the case, the underlined sequence could be a footprint sequence of the *hAT* element. **E)** Comparison of TIR sequences of *hAT* transposons from *Ipomoea purpurea* and *Cuscuta* plants. **F)** Comparison of entire sequences of the *hAT* elements from *Cuscuta* plants. Terminal inverted repeat (TIR) sequences are underlined.

G

141	GTAAATAC**TA**	*CeurSto1* (268 bp)	**TA**TCATTTTG	428
1021	TTGCAAAA**TA**	*CeurSto2* (193 bp)		
1525	TTACCATA**TA**	*CeurSto3* (206 bp)	**TA**	
		CeurSto4 (246 bp)	**TA**TAACACTA	1998
2038	TATTTTAG**TA**	*CeurSto5* (207 bp)	**TA**ATATTTTT	2264
2269	ACCCCATA**TA**	*CeurSto6* (348 bp)	**TA**TATAATAA	2636

H

Stowaway conserved TIR	CTCCCTCCGTT
CeurSto1 5´ TIR	CTCCCTCCGTC
CeurSto1 3´ TIR	CTCCCTCCATC
CeurSto2 5´ TIR	CTCCCTCCCTT
CeurSto2 3´ TIR	
CeurSto3 5´ TIR	CTCCCTCCGTC
CeurSto3 3´ TIR	CTACCTCCGTC
CeurSto4 5´ TIR	CTCCCTCCGTC
CeurSto4 3´ TIR	CTCCCTCCGTC
CeurSto5 5´ TIR	CTCCCTCCGTT
CeurSto5 3´ TIR	CTTCCTCCGTT
CeurSto6 5´ TIR	CTCTCTCCGTT
CeurSto6 3´ TIR	CTCCATCCGTT

I

```
CeurSto2   CTCCCTCCCTTCTTATTTTATCTTCCAAATTGGTTTGTCACACAATTTAAGATAGTGAAT    60
CeurSto5   CTTCCTCCGTTCCTATTTTATCTTCCAAATAGGGTTGGCACACAATTTAAGATAGTGGGT    60
           ** ***** *** ***************** ** *** *******************  *

CeurSto2   ATTGTGTGGTGGAGAGTTGTGTAGAGGAAAGAGAAATGTGTAAGAAATGTTGTGAACCAC   120
CeurSto5   ATTGTGTGGTGGAGAGTTGTGCAGAGGAGAGAGAAATGTGTAAGAAATGTTGTGAACCAC   120
           ********************* ****** *******************************

CeurSto2   TAATTCTTTACTAAAAAGGGAAAGTGAAAGTTTTAATTAGAACAGACGAAAAAGGAAAAT   180
CeurSto5   AAATTCTTTACTAAAAAGGGAAAATGTAAGTTTTAATTGGAACGAACGAAAAAGGAAAGT   180
            ********************** ** *********** ****  ************* *
CeurSto2   TAGAAGTTTTAAT--------------                                   193
CeurSto5   TGGAAGTTTTAATAGGAACGGAGGGAG                                   207
           * ***********
```

Fig. S11. DNA transposons in introns of *Cuscuta CYP81Q* genes (continued)

G) The insertion sites of the *CeurSto* elements. The TA dinucleotide target site duplications are indicated in boldface. *CeurSto3* and *CeurSto4* are adjacent to each other across 2 bp of TA. *CeurSto2* lacks its 3’ terminus. Numerals indicate sequence positions in the intron sequences. **H)** Comparison of the conserved TIR sequences of *Stowaway* elements. **I)** Comparison of entire sequences of *CeurSto2* and *CeurSto5*. *CeurSto2* and *CeurSto5* are inserted in opposite directions, and the complementary sequence of *CeurSto5* is shown. The conserved sequences of the terminal inverted repeats (TIR) are underlined.

J

```
CeurSto7                 ---------CGTCCCGTTGGATTTTGTCAAGTGCAAAGTATGTGAAAAATAAGAATAGTA   51
3002310-3002510          TACTACCTCCGTCCCGGTGGACTTTGCCAAGTGCAAAGTAGGTGAGAAATAAGAATAGTA   60
25554319-25554519        TACTACCTCCGTCCCGGTGGACTTTGCCAAGTGCAAAGTAGGTGAGAAATAAGAATAGTA   60
37127923-37127724        TACTACCTCCGTCCCGGTGGATTTTGCCAAATGCAAAGTAGGTGAGAAATAAGAATAGTA   60
                                  ******* **** **** *** ********* **** **************

CeurSto7                 CGAAGTACAAAGTGTGTGATTAAGATTTGTGTAGAGATGAGAGAAATGTACACAACCATA   111
3002310-3002510          A-------AAACTGTGTGGTTGAGATTTGTGCAGAGGAGAGAGAAATGTGCAGAACCACA   113
25554319-25554519        A-------AAACTGTGTGATTGAGATTTGTGCAGAGGAGAGAGAAATGTGCAGAACCACA   113
37127923-37127724        A-------AAACTGTGTGGTTGAGATTTGTGCAGAGGAGGGAGAAATGTGCAGAACCACA   113
                                 *** ****** ** ********* ****  * ********* ** ***** *

CeurSto7                 AATTAGTTGCTTTAATAGGAAAGTGAGAAGTTTTTTTGTGACAATCAAAAAAGAAAAGTT   171
3002310-3002510          AATTAGTTGTTTTAATAGGAAAGTGACAAAGTTTTTTGGGACAACCAAAAAAGGAAAGTT   173
25554319-25554519        AATTAGTTGCTTTAATAGAAAAGTGCCAAAGTTTTTTGGGACAACAAAAAAAGGGAAAGTT  173
37127923-37127724        AATTAGTTGCTTTCATAGGAAAGTGACAAAAATTTTTGAGACAAC-AAAAAAGGAAAGTT   172
                         ********* *** **** ******  **   ****** *****  ***** * ******

CeurSto7                 GGCAAAGTCCAATGGGACG---------                                   190
3002310-3002510          GGCAAAGTCCAATGGGACGGAGGGAGTA                                   201
25554319-25554519        GGCAAAGTCCAATGGGACGGAAGGAGTA                                   201
37127923-37127724        GGCAATGTCCAATGGGACGGAGGAAGTA                                   200
                         ***** *************
```

Fig. S11. DNA transposons in introns of Cuscuta CYP81Q genes (continued)

J) Comparison of *CeurSto7* and its relative sequences on the *C. europaea* genome sequence (CAMAPE010000005.1). The titles of sequences other than *CeurSto7* are positions in the genomic sequence. *CeurSto7* lacks its TIR sequences. The conserved sequences of the terminal inverted repeats are underlined. The target site duplication sequences are shown in boldface.

K

CAH9094665	180	AGAAATTA**TA**	*CepiSto1* (253 bp)	**TA**TGTGGGTT	452
CAH9141928	180	AGAAATTATA		--TGTGGGTT	197
CAH9094665	1259	TTCACA---------	*CepiSto2* (247 bp)	TAGTTGTGTT	1521
CAH9141928	1386	TTCACA(18 bp)TA		--CTTGTGTT	1419
CAH9094665	1051	GAAGTTGAATTATTAT		---------TGAATGAT	1074
CAH9141928	815	GAAGTTG**AATTATTAT**	*TE like1* (362 bp)	**AATTATTAT**TGAATGAT	1209

L

Stowaway conserved TIR	CTCCCTCCGTT
CepiSto1 5´ TIR	CTTCATCCGTT
CepiSto1 3´ TIR	CTCCCTCCGTT
CepiSto2 5´ TIR	---------TT
CepiSto2 3´ TIR	CTTCCTCTGTT

Fig. S11. DNA transposons in introns of *Cuscuta CYP81Q* genes (continued)

K) Comparison of the intron 2 sequences of *C. epithymum CYP81Q* genes. Shown in bold are target site duplications of 2 bp and 8 bp for *CepiSto1* and *TE like1*, respectively. Numerals indicate sequence positions in the intron sequences. *CepiSto2* is deleted at its 5´ terminus along with its adjacent sequence. **L)** Comparison of the terminal inverted repeat (TIR) sequences of *CepiSto* elements.

M

```
CepiSto2        -----------TTCTTTTTTAATTGCAACATTGGACTTTTTGCACTACTCACATATTCTA   49
972282-972539   TACTACCTCCGTTCTCTTTTAATTGCAACATTGGACTTTTTGCACTATTCACATGTTCTA   60
166873-167132   TACTACCTCCGTTCTCTTTTAGTTGCAACGTTGGACTTTTTGCACTATTCACATATTCTA   60
1374411-1374670 TACTACCTCTGTTCTCTTTTAATTGCAACGTAGGACTTTTTGTACTATTCACACATTCTA   60
                           **** ***** ******* * ********** **** *****  *****

CepiSto2        CTTTGACTATATTTTATTTATTAATGTATTATAAATTAAATTTTTAAAAAATATAGTTAA   109
972282-972539   CTTTGACTATATTTTATTAATTAATGTTTTATAAATTAAAATGTTAAAAAATATTGTTAA   120
166873-167132   CTTTGACTACATTTTATTAATTGATGTATAATAAATTAAATTTTTAAAAAATATTGTTAA   120
1374411-1374670 CTTTGAGTATAGTTTATTTATTAATGTATTACGAATTTAATTTTTAAAAAATATTGTTAA   120
                ****** ** * ****** *** **** * *  **** ** * *********** *****

CepiSto2        ATTATTCACATTCTAAATTTTCGAAATCTGGATTTTTTCAATTTTTTACTAATAAGGAAC   169
972282-972539   ATTCTTCTCATTCTAAATTTTCAAAATATGAATTTTTAAATTTTTTTACTATTAAGGAAT   180
166873-167132   ATTCTTCTCATTCTAAATTTTCAAAATATGAATTTTTTATTTTTTTTACTAATACGGAAT   180
1374411-1374670 ATTCTTCTCATTCTAAATTTTCAAAATATGAATTTTTGAAAATATTTACTAATAAGGATT   180
                *** *** ************** **** ** ******     * ******* ** ***

CepiSto2        GAAAGATGTTAATGATCAAACTTGTGCGTTGGCAAACGTGAAATATTGACTGTTGCAATT   229
972282-972539   GAAAGATATTAATGGTCAAACTTGTGCGTTGGCAAACGT--AATGTTGACTGTTGCAATT   238
166873-167132   GAAAGATATTAATGGTCAAACTTGTGCGTTGGCAAACGTGAAATGTTGACTGTTGCAATA   240
1374411-1374670 GAAAGATATTAATGGTCAAACTCGTGCGTTGACAAACGTGAAATGTTGACTGTTGCAATT   240
                ******* ****** ******* ******** *******  *** **************

CepiSto2        AAAAGAGAACAGAGGAAGTA                                           249
972282-972539   AAAAGGGAACGGAGGAAGTA                                           258
166873-167132   AAAAAGGAACGGAGGAAGTA                                           260
1374411-1374670 AAAAGGGAACGGAGGAAGTA                                           260
                ****  **** *********
```

N

```
TE like1 5´ TIR    GGAAAAATTGATTATAATAATCCCAACTATTGACGGTCCGCTTATAATAATCTCAACTGT   60
TE like1 3´ TIR    GGAAAAATCGAAAATAATAATCCCAACTATTACCCATCCGCTAACATTAATCCCAACTAT   60
                   ******** **  ******************  *  ****** * * ***** ***** *

TE like1 5´ TIR    CGATTATTTCAGAATAATCCCAACTTTAGGGACCTTCTGCCAATAATAGTCC           112
TE like1 3´ TIR    TGATTATTTTTGTATAATCCCAACTATATGGGTCATCCGCCAATAATGGTCC           112
                    ********  * ************ ** **  * ** ********* ****
```

Fig. S11. DNA transposons in introns of *Cuscuta CYP81Q* genes (continued)

M) Comparison of *CepiSto2* and its relatives on the *C. epithymum* genome sequence (CAMAPF010000082.1). The sequence titles other than *CepiSto2* are positions in the genomic sequence. *CepiSto2* lacks its 5´ TIR. The conserved sequences of the terminal inverted repeats are underlined. The target site duplication sequences are shown in boldface. **N)** The terminal inverted repeats (TIR) sequences of *TE like1*.

O

```
Cchi_CYP81Q2 intron 2
2,260 AATTGTAATA      CchiSto1   TATTACTTTA      2541
3,147 GAATATAATA      CchiSto2   TATTAGATTA      3382
3,682 TAAGATAGTT      CchiSto3
```

P

```
Stowaway conserved TIR    CTCCCTCCGTT
CchiSto1 5´ TIR           CTCCCTCTGTC
CchiSto1 3´ TIR           CTCCCCCCGTC
CchiSto2 5´ TIR           TTACCTCCGTC
CchiSto2 3´ TIR           CTCCCTCCGTC
CchiSto3 5´ TIR           CTCCCTCCATC
```

Q

```
CchiSto4   3825  GGAAAGTGGAACCTTTCTTTGCGACAAACAAAAAAGAAAA  3864
CceurSto7  129   GGAAAGTGAGAAGTTTTTTTGTGACAATCAAAAAAGAAAA   168
                 ********  *  *** **** ***** ************
```

Fig. S11. DNA transposons in introns of *Cuscuta CYP81Q* genes (continued)

O) The target sites of the *Stowaway* elements in intron 2 of *CchiCYP81Q-2*. The TA dinucleotide target site duplication is indicated in boldface. Numerals indicate sequence positions in the intron sequences. **P)** Comparison of the conserved terminal inverted repeat (TIR) sequences of *Stowaway* elements. **Q)** Comparison of entire sequences of the partial *Stowaway* elements of *CchiSto4* and *CeurSto7*. Numerals indicate the position within the intron for *CchiSto4*, and for *CeurSto7*, it denotes its position within its own sequence.

R

```
Cchi_CYP81Q2          228         TATGGGTAAAGTAAT CchiLTR1   ATGTTTTTTAAGCGG    953
CcLTR1 scaffold24     875183      TTGTTTGACATATAC CcLTR1     TATACTTTCTCTTGA 874458
```

S

```
CcLTR1      TGTAACACCCCGAC---CCCCACCCTAG-CCGTAATTGTCCGCTCATAGGATTGTAGATT      56
CchiLTR1    TGTAACACTCTAGCTAGCCCCACCCCAGGCCGGTAATGACTACTCATGAGATTATAGGTT      60
            ******** *   *   ******** ** ***  * ** *  *****  **** *** **

CcLTR1      GGCCCCACAAACCAACACAAGTCTTTTCCGCGCACTTTGTCCTCACTCGTGCGCGCCCAA     116
CchiLTR1    GGCCCTACAATTCAACACAAGATTTTCTCGTGCATTTTGGCCTCACTCATGCACGCTCT-     119
            ***** ****  *********  ***  ** *** **** ******** *** *** *

CcLTR1      GGAAACTTCCCAGGGGGTCACCCATCCTAGAATTGCTCCAAGTCAAACACGCTTAACTTT     176
CchiLTR1    GAAAACTTCCCAGGGAGTCGCA-ATCCTAGAATTGCTCCAAATTAAACATGCTTCACCTT     178
            * ************* *** *  ****************** * ***** **** ** **

CcLTR1      GGAGTTTTTAGGTGATGAGCTCTCGAAAAGAAGATGCACCTTGTTGGTAT--GAGTAGTA     234
CchiLTR1    GGAGTTTTTAAGTTATGAGCTCTTGAAAAGAAGGTGCATCTTGTTGATATACGAGTAGTA     238
            ********** ** ********* ********* **** ******* ***  ********

CcLTR1      CTATCAATCCATTTGAGCTCTTCTCATTC----TCATCTTAACACAGGGTATTACATTCA     290
CchiLTR1    TAATCAATTTTTTTGAGTTCTTTTCAATCCATCTCATTTCAACACGGGATATTACATTCA     298
              ******   ****** **** *** **    **** * ***** ** ***********

CcLTR1      CCCCCACTTAGGAATGCAACGTCCTCGTTGCATCCTTTTTTTAAACCCCTAGATCTTCCC     350
CchiLTR1    CCCTCACTTAAGAATACAACATCCTCGTTGCAC-----------GCCCCAAGATCCTCCC     347
            *** ****** **** **** ***********             **** ***** ****

CcLTR1      C-GCTAGGTGAACCAAGCTCTTCTTGGCCCGTCACGGTCGCAGGGTTGCTCTGATACCAT     409
CchiLTR1    CCACTAGGTGAACTGAGCTCGT--TGGCCCATCACAACCGCAAAATTGCTTTGATACCAT     405
            *  ********** ***** * ****** ****   ****   ***** *********

CcLTR1      TTGTAACACCCAGGCCCCCACCCTAGCCGTAAGTGTCCGCTCATAGGATTGTAGACTGGC     469
CchiLTR1    TTGTAACACTTTGGCCACCCCTCGGGCCAGAAGTGACTACTCATGAGATTGTAAACTTGC     465
            *********   **** ** * *  ***  ***** *  *****  ******* *** **

CcLTR1      CCCACAAACCAACACAAGTCTTTTCCGCGCACTTTGTCCTCACTCGTGCGCGCCCAAAGA     529
CchiLTR1    CTCACGAACCAACACAAGTCTTTCCCGCGCATTTTAGCCTCATTCATGTGCGCCTTGA-A     524
            * *** ***************** ******* ***  ***** ** ** *****   * *

CcLTR1      AACTTCTCAGGGGGTCACCCATCCTAGAATTGCTCCAGGTCAAGCACGCTTAACTTTGGA     589
CchiLTR1    AACATTCCAGGGGGTCACCAATCATAGAATTGCTCCAAGTCAAGCATGTTTTACTTTGGA     584
            *** *  ************ *** ************* ******** * ** ********

CcLTR1      GTTTCTAGGTGATGAGCTCTCGAAAAGAAGATGCACCTTGTTGGTATGAGTAGTACCATC     649
CchiLTR1    GTTTCTAAGTTATGGACTCTCAAAAATTAGATGCATCTTGTTGGTAAAAGTGGTACTATA     644
            ******* ** ***  ***** ****  ******* **********  *** **** **

CcLTR1      AATCCATTTA--AGCTCTTCTCAT---TCTCATCTTAACACAGGGTATTACA             696
CchiLTR1    AATCCTTTTTTTAGTTCTTTCAATCCATCTCATTTCAATTCGGAGTATTACA             696
            ***** ***   ** ****   **   ****** * **  * * ********
```

Fig. S11. DNA transposons in introns of *Cuscuta CYP81Q* genes (continued)

R) The target sites of the retrotransposons, *CcamLTR1* and *CchiLTR1*. *CcamLTR1* is identified in the *C. campestris* genome assembly, Ccam0.32_scaffold24 (GenBank accession number: OOIL02001568.1). The putative 5-bp target site duplication is highlighted in boldface. Numerals denote sequence positions in the intron sequences and the scaffold sequence for *CcamLTR1* and *CchiLTR1*, respectively. **S)** A comparison of the entire sequences of the retrotransposons is shown, with the Long Terminal Repeat (LTR) sequences being underlined.

Supplementary Method

Estimation of divergence time in *Cuscuta* species

Since previous analyses of dodders predicted that *C. chinensis*, *C. campestris* and *C. epithymum* are polyploids, we treated their genomes as two homoeologous subgenomes (Ibiapino et al., 2022; Neumann et al., 2023; Segawa et al., 2026). To estimate the divergence times within *Cuscuta*, protein sequences of *C. epithymum* were subjected to a self–BLASTP search for each (sub)genomes, and the resulting homology information was used to identify syntenic contigs with MCScanX (Wang et al. 2012). Syntenic contigs identified using MCScanX were similarly classified into subgenomes. Using OrthoFinder (Emms and Kelly 2019), we identified 472 single-copy orthologous genes shared across the proteomes of *C. australis*, *C. campestris* (BI and BII), *C. chinensis* (HI and HII), *C. epithymum* (A and A'), *C. europaea*, *Ipomoea triloba* (GCF_003576645.1), *I. nil* (Asagao_1.2 genome, http://viewer.shigen.info/asagao/), *S. lycopersicum* (GCF_000188115.5), *S. indicum* (GCA_000512975.1), and *A. thaliana* (Araport11_pep_20220914 from TAIR, https://www.arabidopsis.org/). The amino acid sequences of the single-copy genes were aligned using MAFFT (--maxiterate 1000 --localpair) (Katoh et al. 2005), and the resulting alignments were then converted to nucleotide sequences using PAL2NAL version 14.1 (Suyama et al. 2006). From these alignments, 6649 fourfold degenerate third-codon transversion (4DTv) were extracted for divergence time estimation using BEAST version 2.7.5 (Bouckaert et al. 2019). BEAUti was configured with the following parameters: Substitution Rate = "Tree" (estimated); Gamma Category Count = 4; Shape = estimated; Substitution Model = GTR; Clock Model = Relaxed Clock Log Normal; MCMC Chain Length = 20,000,000. The calibration points were set as described below: The divergence between *A. thaliana* (Brassicales) and Solanales: 85.80–128.63 Mya (Morris et al. 2018); The divergence between Solanaceae and Convolvulaceae: 59.1–83.9 Mya (Eserman et al. 2014); The divergence between *Cuscuta* and *Ipomoea*: 47.8-56.0 Mya (Muller 1981).

Supplementary References

Bouckaert R, Vaughan TG, Barido-Sottani J, Duchêne S, Fourment M, Gavryushkina A, Heled J, Jones G, Kühnert D, De Maio, N, *et al.* BEAST 2.5: An advanced software platform for Bayesian evolutionary analysis. *PLoS Comput. Biol.* 2019:15: e1006650. DOI: 10.1371/journal.pcbi.1006650

Emms DM, Kelly S. OrthoFinder: phylogenetic orthology inference for comparative genomics. *Genome Biol.* 2019:20:238. DOI: 10.1186/s13059-019-1832-y

Eserman LA, Tiley GP, Jarret RL, Leebens-Mack JH, Miller RE. Phylogenetics and diversification of morning glories (tribe Ipomoeeae, Convolvulaceae) based on whole plastome sequences. *Am. J. Bot.* 2014:101:92-103. DOI: 10.3732/ajb.1300207

García MA, Costea M, Kuzmina M, Stefanović S. Phylogeny, character evolution, and biogeography of *Cuscuta* (dodders; Convolvulaceae) inferred from coding plastid and nuclear sequences. *Am. J. Bot.* 2014:101:670–690. https://doi.org/10.3732/ajb.1300449

Gouy M, Guindon S, Gascuel O. SeaView version 4: A multiplatform graphical user interface for sequence alignment and phylogenetic tree building. *Mol. Biol. Evol.* 2010:27:221–224. https://doi.org/10.1093/molbev/msp259

Grabherr MG, Haas BJ, Yassour M, Levin JZ, Thompson DA, Amit I, Adiconis X, Fan L, Raychowdhury R, Zeng Q, *et al.* Full-length transcriptome assembly from RNA-Seq data without a reference genome. *Nat. Biotechnol.* 2011:29:644-652. https://doi.org/10.1038/nbt.1883

Ibiapino A, García MA, Amorim B, Baez M, Costea M, Stefanović S, Pedrosa-Harand A. The Evolution of Cytogenetic Traits in Cuscuta (Convolvulaceae), the Genus with the Most Diverse Chromosomes in Angiosperms. *Front. Plant Sci.* 2022:13:842260. doi:10.3389/fpls.2022.842260

Kaga Y, Yokoyama R, Sano R, Ohtani M, Demura T, Kuroha T, Shinohara N, Nishitani K. Interspecific signaling between the parasitic plant and the host plants regulate xylem vessel cell differentiation in haustoria of *Cuscuta campestris*. *Front. Plant Sci.* 2020:11:193. https://doi.org/10.3389/fpls.2020.00193

Katoh K, Kuma K, Toh H, Miyata T. MAFFT version 5: improvement in accuracy of multiple sequence alignment. *Nucleic Acids Res.* 2005:33:511–518. https://doi.org/10.1093/nar/gki198

Kim HJ, Ono E, Morimoto K, Yamagaki T, Okazawa A, Kobayashi A, Satake H. Metabolic engineering of lignan biosynthesis in *Forsythia* cell culture. *Plant Cell Physiol.* 2009:50:2200-2209. https://doi.org/10.1093/pcp/pcp156

Kurtz S, Phillippy A, Delcher AL, Smoot M, Shumway M, Antonescu C, Salzberg SL. Versatile and open software for comparing large genomes. *Genome Biol.* 2004:5:R12. https://doi.org/10.1186/gb-2004-5-2-r12

Morris JL, Puttick MN, Clark JW, Edwards D, Kenrick P, Pressel S, Wellman CH, Yang Z, Schneider H, Donoghue PCJ. The timescale of early land plant evolution. *Proc. Natl. Acad. Sci. U. S. A.* 2018:115: E2274-E2283. DOI: 10.1073/pnas.1719588115

Muller J. Fossil pollen records of extant angiosperms. *Bot. Rev.* 1981:47:1.

Neumann P, Oliveira L, Jang TS, Novák P, Koblížková A, Schubert V, Houben A, Macas J. Disruption of the standard kinetochore in holocentric Cuscuta species. *Proc. Natl. Acad. Sci. U. S. A.* 2023:120:e2300877120. doi: 10.1073/pnas.2300877120

Ono E, Nakai M, Fukui Y, Tomimori N, Fukuchi-Mizutani M, Saito M, Satake H, Tanaka T, Katsuta M, Umezawa T, *et al.* Formation of two methylenedioxy bridges by a Sesamum CYP81Q protein yielding a furofuran lignan, (+)-sesamin. *Proc. Natl. Acad. Sci. U. S. A.* 2006:103:10116–10121. https://doi.org/10.1073/pnas.0603865103

Segawa T, Yoshizumi S, Toyonaga H, Shiraishi A, Sato K, Yamabe T, Takagi M, Takagawa M, Yokoyama R, Itoh T, *et al*. Chromosome-scale genome assemblies of two allopolyploid *Cuscuta* species uncover genomic signatures of parasitic lifestyle and polyploid evolution. *Plant Cell Physiol.* 2026:pcag002. https://doi.org/10.1093/pcp/pcag002

Sun G, Xu Y, Liu H, Sun T, Zhang J, Hettenhausen C, Shen G, Qi J, Qin Y, Li J, *et al.* Large-scale gene losses underlie the genome evolution of parasitic plant *Cuscuta australis*. *Nat. Commun.* 2018:9:2683. https://doi.org/10.1038/s41467-018-04721-8

Suyama M, Torrents D, Bork P. PAL2NAL: robust conversion of protein sequence alignments into the corresponding codon alignments. *Nucleic Acids Res.* 2006:34(Web Server issue):W609-12. doi: 10.1093/nar/gkl315

Vogel A, Schwacke R, Denton AK, Usadel B, Hollmann J, Fischer K, Bolger A, Schmidt MH-W, Bolger ME, Gundlach H, *et al.* Footprints of parasitism in the genome of the parasitic flowering plant *Cuscuta campestris*. *Nat. Commun.* 2018:9:2515. https://doi.org/10.1038/s41467-018-04344-z

Wang Y, Tang H, Debarry JD, Tan X, Li J, Wang X, Lee T, Jin H, Marler B, Guo H, *et al.* MCScanX: a toolkit for detection and evolutionary analysis of gene synteny and collinearity. *Nucleic Acids Res.* 2012:40:e49. https://doi.org/10.1093/nar/gkr1293

Xiang, Sheng-Xi; He, Zhi-Sheng; Ye, Yang. Furofuran lignans from Cuscuta chinensis. Chinese J. Chem. 2001: 19: 282-285. https://onlinelibrary.wiley.com/doi/pdf/10.1002/cjoc.20010190314

Yahara S, Domoto H, Sugimura C, Nohara T, Niiho Y, Nakajima Y, Ito H. An alkaloid and two lignans from *Cuscuta chinensis*. *Phytochem.* 1994:37:1755-1757. https://www.sciencedirect.com/science/article/abs/pii/S0031942200896053?via%3Dihub

Other supporting materials for this manuscript

Table S1. Identifiers of proteins used in the phylogenetic analysis in Figure 3

Table S2. Comparison of *CYP81Q* genes and *CYP81*-related genes to *CcamCYP81Q111* and *SiCYP81Q1*

Table S3. Nucleotide sequences of exon 2 of *CYP81Q* genes and *CYP81*-related genes

Table S4. Deduced amino acid sequences of *CYP81Q* genes and *CYP81*-related genes

Table S5. Genomic synteny among *Cuscuta* and related species

Table S6. Transposons in *Cuscuta CYP81Q* genes

Table S7. Genome assembly containing Gene Feature Files used in the analysis of the number of introns per gene

Table S8. Nucleotide sequences of the coding regions of the *CYP81Q* genes and *CYP81*-related genes

Table S9. Sequences of oligonucleotide primers and CDSs used in this study
